# Recent advances in Ti_3_C_2_T_*x*_ MXenes for photonic applications

**DOI:** 10.1039/d6na00163g

**Published:** 2026-08-25

**Authors:** Thi Thu Trinh Phan, Qui Thanh Hoai Ta, Phan Khanh Thinh Nguyen, Ly Tan Nhiem

**Affiliations:** a Chemistry and Biochemistry Department, Utah State University 300 Old Main Hill Logan UT USA; b Institute of Advanced Technology, Vietnam Academy of Science and Technology 1B TL29 Street, An Phu Dong Ward Ho Chi Minh City 700000 Vietnam; c School of Chemical, Biological, and Battery Engineering, Gachon University 1342 Seongnamdaero, Sujeong-gu Seongnam-si Gyeonggi-do 13120 Republic of Korea thinhnpk@gachon.ac.kr; d Faculty of Chemistry and Life Sciences, Ho Chi Minh City University of Technology and Engineering 01 Vo Van Ngan Street, Thu Duc Ward Ho Chi Minh City Vietnam nhiemlt@hcmute.edu.vn

## Abstract

In recent years, Ti_3_C_2_T_*x*_ MXenes have emerged as highly promising materials due to their unique physicochemical properties, including mechanical, electronic, magnetic, and optical characteristics, as well as their ease of synthesis. This review provides a comprehensive overview of the role of Ti_3_C_2_T_*x*_ MXenes in photonic technologies. MXenes demonstrate strong nonlinear optical absorption, enabling the generation of ultrafast laser pulses across a broad spectral range from the visible to the mid-infrared regions. Furthermore, their intrinsic metallic conductivity facilitates long-wavelength applications, particularly in Ti_3_C_2_T_*x*_-based saturable absorber mirrors. The relationship between photovoltaic efficiency and the material's optical properties and bandgap energy is also discussed. Recent advances in MXene-based heterostructures suggest strong potential for scalable integration into photonic technologies, including nonlinear optical devices, transparent conductive electrodes, photovoltaics, and plasmonic systems, although challenges related to stability and interface control persist.

## Abstract 2

In recent years, Ti_3_C_2_T_*x*_ MXenes have emerged as highly promising materials due to their unique physicochemical properties, including mechanical, electronic, magnetic, and optical characteristics, as well as their ease of synthesis.

## Graphical abstract

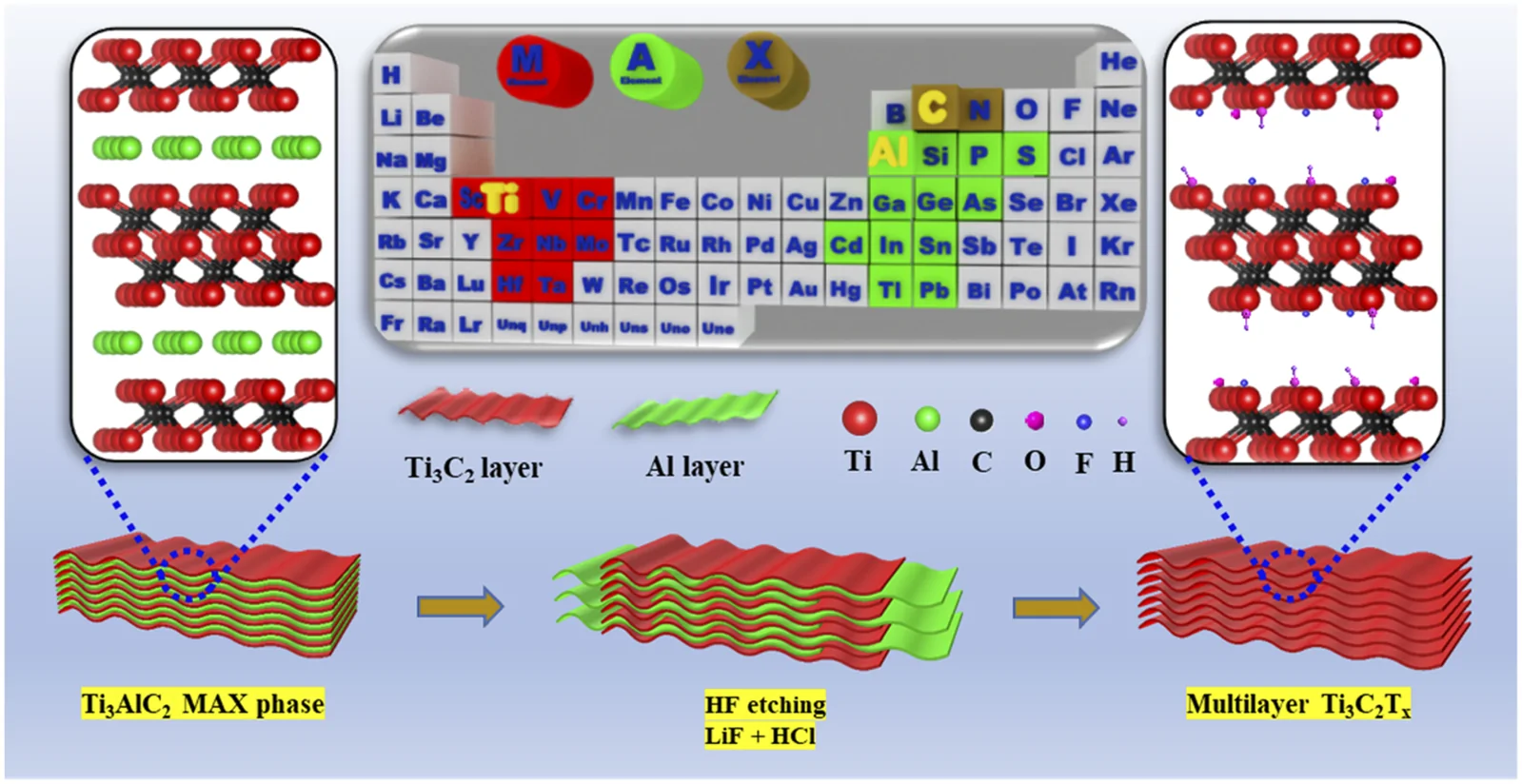

## 1. Introduction

Since their initial exploration in 2011, Ti_3_C_2_T_*x*_ MXenes have attracted substantial attention from the scientific community across many fields, including sensors, energy storage, photonics, electronics, and environmental applications.^1–5^ This interest is owing to their electrical conductivity, good mechanical properties, natural hydrophilicity, and large surface areas. MXenes are two-dimensional (2D) carbonitrides, transition-metal carbides, and nitrides.^6,7^ They are typically synthesized by etching the MAX phase in an HF solution or through an *in situ* HF procedure. The bulk MAX phase possesses a layered hexagonal structure with the space group P6/mmc and is represented by the formula M_*n*+1_AX_*n*_, where M refers to an early transition metal, A denotes an element from groups IIIA–VA, and X represents N and/or C (*n* = 1–4). Although the covalent/ionic M–X bonding in the MAX phase is stronger than that of the metallic M–A bonding, it can be easily broken with a suitable etchant, resulting in the formation of M_*n*+1_X_*n*_T_*x*_ MXenes, where T_*x*_ represents the termination group (–O, –F, –OH, –Br, or –S) that depends on the etching agent used. To date, more than 40 types of MXenes have been theoretically and experimentally prepared.^8–11^ The lower valency of the A elements and M atom, combined with the larger atomic radii of the A elements, results in higher exfoliation energy. Consequently, the Ti_3_AlC_2_ MAX phase is typically transformed into a Ti_3_C_2_T_*x*_ MXene upon etching the aluminum layers under comparatively mild conditions. For instance, at 40 °C, Ti_3_SiC_2_ requires 45 hours of etching with a high exfoliation energy of approximately 0.186 eV Å^−2^ in a solution of 30% HF and H_2_O_2_, whereas Ti_3_AlC_2_ requires only 24 hours of etching in a 5% HF solution with an exfoliation energy of 0.164 eV Å^−2^ under mild conditions.^12^

Moreover, Ti_3_C_2_T_*x*_ has been confirmed to possess several unique properties, including high stability and a strong nonlinear optical response.^13–17^ One of its most distinctive features is its metallic conductivity, making it highly suitable for supporting surface plasmon polaritons. Based on their physicochemical properties, MXenes are expected to overcome the existing limitations of current 2D materials, such as limited surface functionalization, low electrical conductivity, high hydrophobicity, and poor biocompatibility.^18^ In a previous study, the integration of noble metals with MXene-based fibers effectively prevented metal oxidation and enhanced the bonding between silica and the metal, thereby maintaining sensing performance for over one year with a high sensitivity of approximately 5000 nm per RIU.^19^ Xiang *et al.* demonstrated that a surface plasmon resonance (SPR) sensor utilizing MXene as a plasmonic material exhibited a performance approximately seven times greater than that of conventional SPR sensors.^20^

The monolayer MXene was demonstrated to be a highly efficient saturable absorber, attributed to its surface plasmon resonance effect. The plasmon-driven relaxation mechanism involves hot electrons, which facilitate ultrafast nonlinear optical responses.^21^ Moreover, MXene exhibits a high saturable absorption coefficient of approximately −1.35 cm GW^−1^. It was demonstrated that ultrasonication effectively tunes the nonlinear scattering properties of S–Ti_3_Al_1−*x*_C_2_, yielding a nonlinear absorption coefficient of approximately −2.4844 cm GW^−1^.^22^ Notably, after thermal treatment at 80 °C for more than 24 h, the sample retained over 80% of its saturable absorption performance. This enhanced stability is attributed to the Al content, which suppresses oxidation.

Researchers are enthusiastically discovering the promising potential of Ti_3_C_2_T_*x*_ in a wide range of applications, including nonlinear photonic devices, transparent conductive electrodes, photovoltaics, and plasmonic photonic systems. Ongoing studies continue to focus on improving the intrinsic properties of Ti_3_C_2_T_*x*_, gaining deeper insights into its fundamental mechanisms, and combining it with other materials to fully unlock its potential for next-generation photonic technologies.

In this review, we focus on recent progress and remaining challenges in the enhancement of Ti_3_C_2_T_*x*_ MXene-based composites for potential applications. First, the synthesis methods, along with the electrical, optical, and mechanical properties of Ti_3_C_2_T_*x*_ and its related heterostructures, are comprehensively discussed. We then summarize recent advances in the photonic applications of MXenes, highlighting their roles in various optoelectronic devices. Finally, future perspectives and a roadmap for improving the performance, scalability, and practical implementation of MXene-based photonic technologies are presented.

## 2. Synthesis methods of Ti_3_C_2_T_*x*_

The selective removal of A layers from MAX phases occurs because the bonding between M–X (covalent or ionic bonding) is stronger than that of M–A (metallic bonding), enabling successful etching while preserving the M_*n*+1_X_*n*_ framework. Notably, carbide MAX phases generally exhibit higher chemical stability and are more extensively investigated than nitride MAX phases, contributing to the broader application of Ti_3_C_2_T_*x*_ MXenes.^23–26^ Furthermore, oxidizer–ligand pairs play a crucial role in regulating the etching process and surface terminations of MXenes.

In the HF-based etching technique, concentrated hydrofluoric acid (HF) directly reacts with the MAX phase to selectively remove the A element and synthesize MXene. Parameters such as HF concentration, reaction temperature, etching duration, and post-treatment washing significantly affect the structural quality, surface chemistry, and defect density of the resulting MXene materials. This method is relatively simple and cost-effective for the preparation of multilayered MXene, and it typically follows the procedural steps outlined below:Ti_3_AlC_2_ + 3HF → AlF_3_ + 1.5H_2_ + Ti_3_C_2_T_*x*_Ti_3_C_2_T_*x*_ + 2HF → Ti_3_C_2_F_2_ + H_2_Ti_3_C_2_T_*x*_ + 2H_2_O → Ti_3_C_2_(OH)_2_ + H_2_

**Fig. 1:**
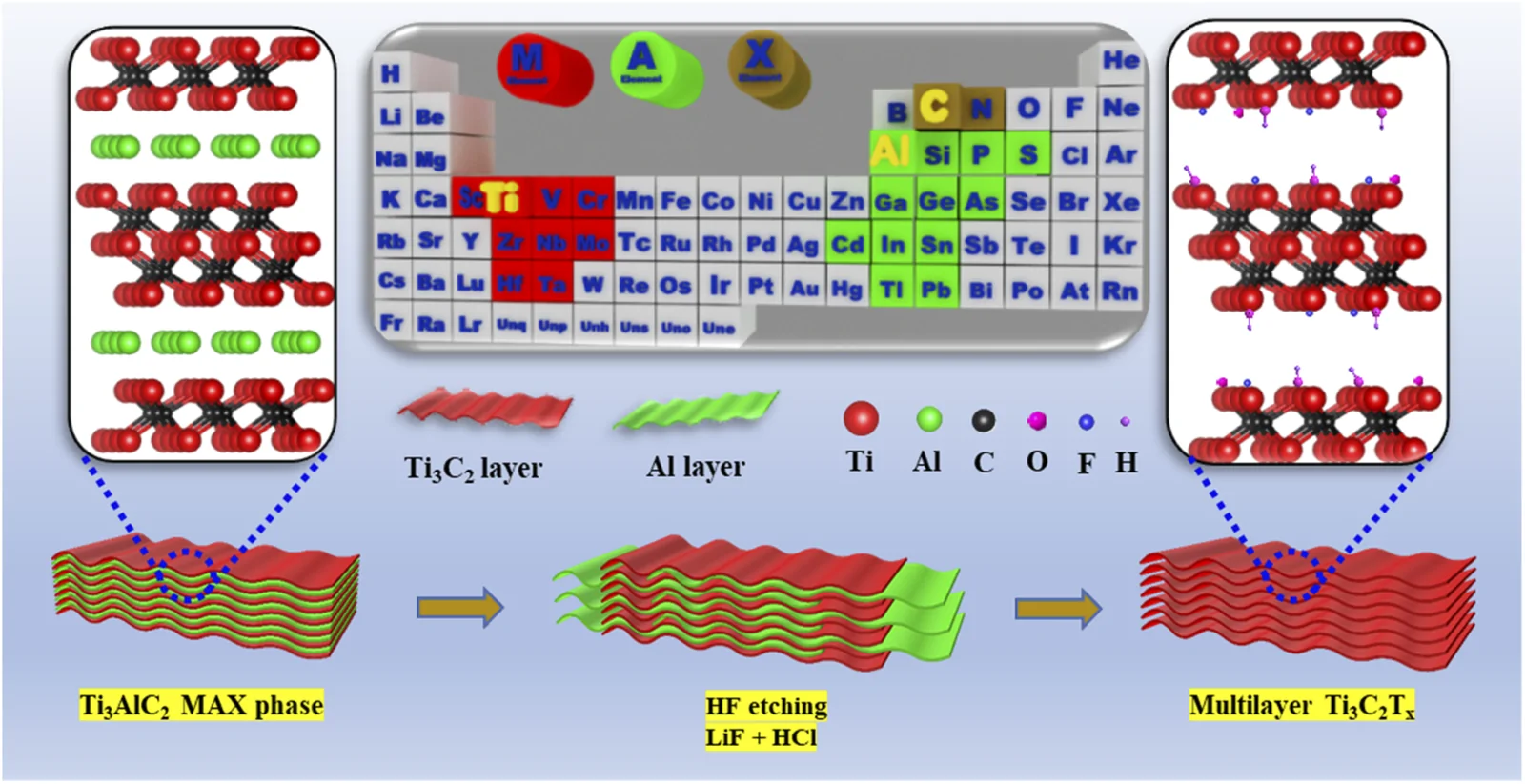
Methodologies for the synthesis of Ti_3_C_2_T_*x*_ MXenes utilizing a fluoride-based approach.^33^ This figure has been adapted/reproduced from ref. 31 with permission from Elsevier, copyright 2021.

In a milder approach, fluoride salts (*e.g.*, KF, LiF, NaF, CsF, and CaF_2_) react with hydrochloric acid (HCl) to generate hydrofluoric acid (HF) *in situ* for the etching process (Fig. 1). During etching, the aluminum (Al) layer is selectively removed and detached from the polycrystalline MAX phase. Although this method is generally considered less efficient than direct HF etching due to fewer available surface-active sites, it offers a significant advantage in yielding high-quality, delaminated single-layer MXene. This is primarily attributed to the effective intercalation of Li^+^ ions, which facilitates impulsive delamination. The surface termination groups of MXenes can be effectively tailored by selecting appropriate etching routes and post-treatment conditions. The type and distribution of surface functional groups are strongly influenced by the etchant chemistry as well as subsequent thermal treatment under either oxidative or inert atmospheres. For example, –OH terminations can be removed through vacuum annealing at elevated temperatures (approximately 773 K, 40 h).^27^ Alternatively, –OH groups may be gradually converted into –O terminations during thermal treatment in the temperature range of 473–1073 K. Under an inert Ar atmosphere, oxygen-containing terminations are preferentially removed at high temperatures,^28^ whereas fluorine terminations exhibit significantly greater thermal stability and are more difficult to detach, even above 1073 K.^29–32^

**Fig. 2:**
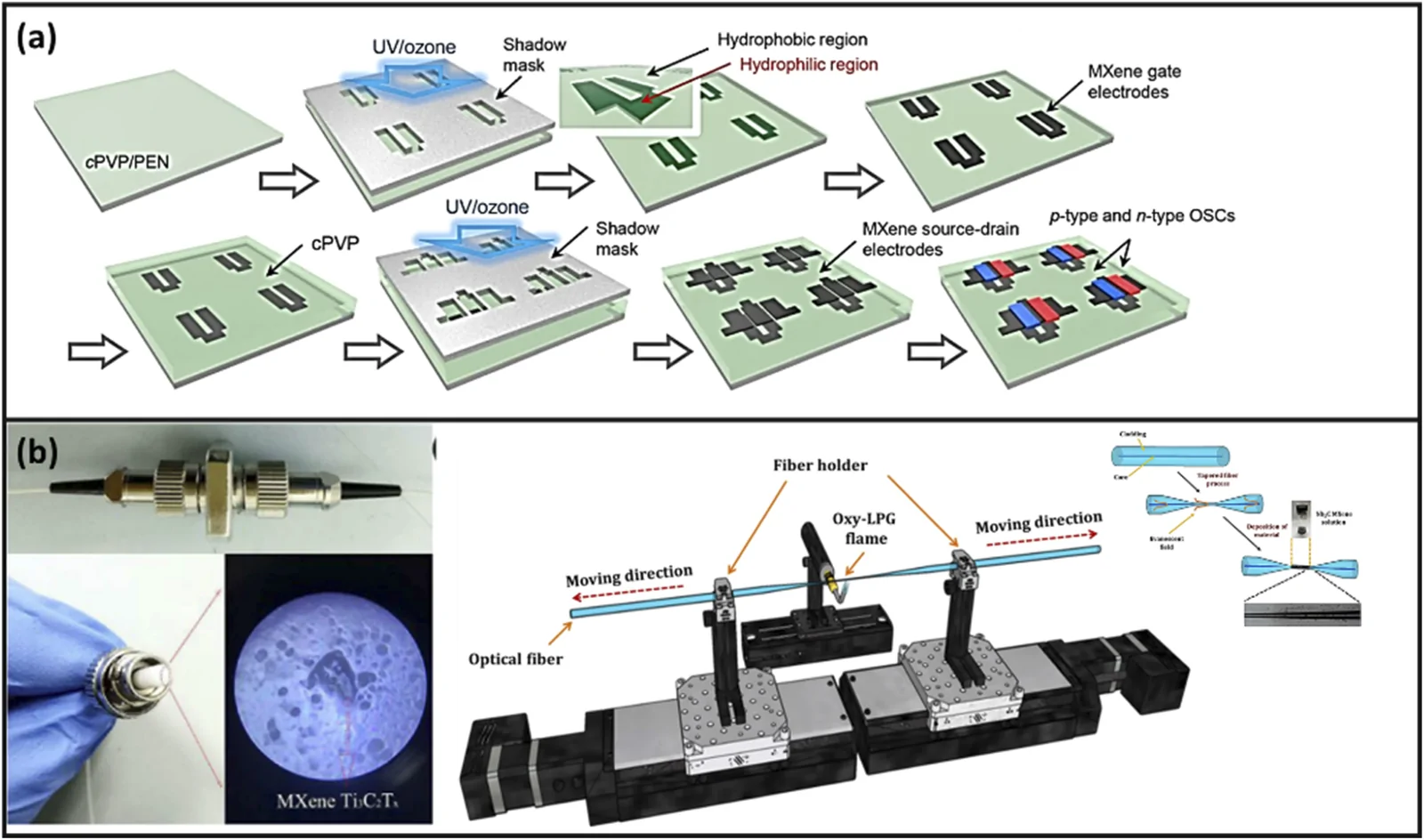
(a) Schematic illustrating the shadow-mask-assisted fabrication steps of OFETs with MXene electrodes.^36^ (b) Photograph of a fiber coated with an MXene and the corresponding saturable absorber (SA) device.^39,40^ This figure has been adapted/reproduced from ref. 39 with permission from Elsevier, copyright 2015.

In addition to the top-down strategy starting from the MAX phase, bottom-up techniques have also been employed to synthesize high-quality ultrathin Ti_3_C_2_T_*x*_ MXenes, including chemical vapor deposition, RF sputtering, ion beam sputtering, and ion irradiation.^34,35^ It is suggested to adopt a strategy that is capable of controlling the functional groups, which directly affect the optical, mechanical, and thermal properties. Furthermore, oxidation resistance needs to be controlled to avoid device degradation. Optimizing these two challenges could improve and expand the long-term operation of Ti_3_C_2_T_*x*_ MXenes in photonic applications. In flexible electronics, Ti_3_C_2_T_*x*_ MXenes serve as the gate, source, and drain electrodes in organic field-effect transistors. The electrodes were patterned using a shadow mask, and the poly(ethylene 2,6-naphthalate) substrate was immersed in an aqueous Ti_3_C_2_T_*x*_ MXene solution. The root-mean-square roughness of Ti_3_C_2_T_*x*_ reached 4.3 nm, with a high conductivity of approximately 2.6 kS cm^−1^ (Fig. 2).^36^ It has been demonstrated that spray-coating and spin-coating methods can also be employed to fabricate MXene electrical contacts on the substrate.^37,38^

## 3. Properties of Ti_3_C_2_T_*x*_

### 3.1. Thermal/chemical stability

**Fig. 3:**
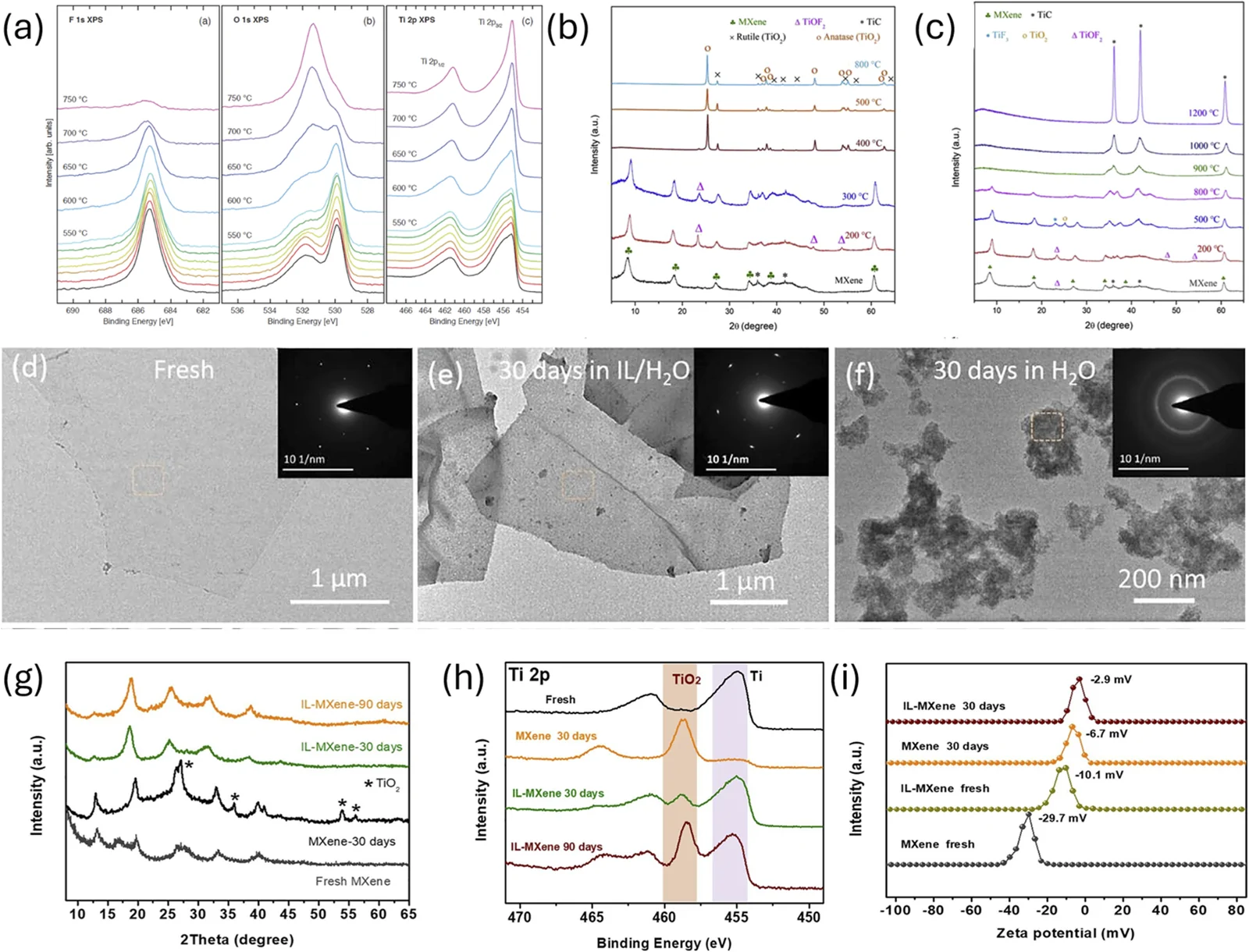
(a) TP-XPS measurements revealing the temperature-dependent behavior of (i) F 1s, (ii) O 1s, and (iii) Ti 2p.^41^ © 2017 IOP Publishing Ltd XRD patterns of Ti_3_C_2_ nanosheets before and after heat treatment in (b) air and (c) vacuum at different temperatures.^43^ Copyright 2014 Elsevier. TEM images and the corresponding selected area electron diffraction (SAED) pattern of MXenes and IL-MXene flakes: (d) fresh MXene, (e) 30 days in 0.1 M IL, and (f) 30 days in water.^49^ (g) XRD patterns, (h) XPS results and (i) zeta potentials of the MXene and IL-MXenes after being stored for different times; peaks of rutile are marked with asterisks.^49^ © 2020 Elsevier.

The thermal stability of MXene was demonstrated by monitoring the decomposition of the sample during thermal treatment in vacuum. The thermal treatment involved removal of surface terminals and phase transformation of the 2D MXene sheets, along with the detachment of physically adsorbed H_2_O molecules at low temperatures. X-ray photoelectron spectroscopy (XPS) provided information on the changes in the functional groups of Ti_3_C_2_T_*x*_ at high temperatures up to 750 °C. At 550 °C, F- groups desorbed, resulting in a decrease in the F 1s peak intensity; moreover, the Ti 2p peaks shifted from 455.9 eV to 455.1 eV due to charge transfer back to the Ti atoms after the desorption of the –F groups (Fig. 3a).^41,42^

Fig. 3b shows the XRD spectra of samples heat-treated for 30 min at temperatures from 200 to 800 °C. The spectra reveal that the recorded diffraction peaks did not change after calcination at 300 °C, but the corresponding peaks of TiOF_2_ appeared. Some other experimental results have also been reported for the formation of TiOF_2_ and TiO_2_ after heat treatment at 200 °C for Ti_3_C_2_T_*x*_ MXene samples synthesized by HF etching at 60 °C for 24 hours.^43^ After heat treatment above 900 °C in vacuum, the XRD spectrum shows that the diffraction peaks of Ti_3_C_2_ completely disappear, and broadened peaks corresponding to TiC appear, indicating the phase transformation of 2D Ti_3_C_2_ nanosheets into TiC (Fig. 3c). At temperatures from 900 °C to 1200 °C, the diffraction peaks of TiC are enhanced and sharper due to the growth of TiC particles, which proves that MXene can be stable at 800 °C in vacuum.^42,44^

Typical TEM images of Ti_3_C_2_T_*x*_ nanosheets with 2–3 µm lateral flake size show a relatively flat surface (Fig. 3d–f) for the pure MXene sample, while this surface changes for samples after 30 days of immersion in 0.1 M aqueous solution of [C_2_NH_2_MIm][Cl] (IL) and after 30 days of immersion in water. Selected area electron diffraction (SAED) pattern (insert of Fig. 3d) shows a hexagonal atomic arrangement, indicating that the nanosheet is single crystalline.^45^ For the MXene sample stored in water after 30 days, the 2D nanosheet morphology could not be clearly observed; MXene was easily decomposed into amorphous TiO_2,_ and the order in the carbon structure was lost.^46^ Meanwhile, the crystal of IL-MXene preserved after aging for 30 days still showed a monolayer and single-crystal nature. Although some TiO_2_ nanoparticles were formed on the flakes, the 2D sheet structure was basically retained, indicating that IL could delay the decomposition of Ti_3_C_2_T_*x*_ sheets.^47,48^

### 3.2. Mechanical properties

**Fig. 4:**
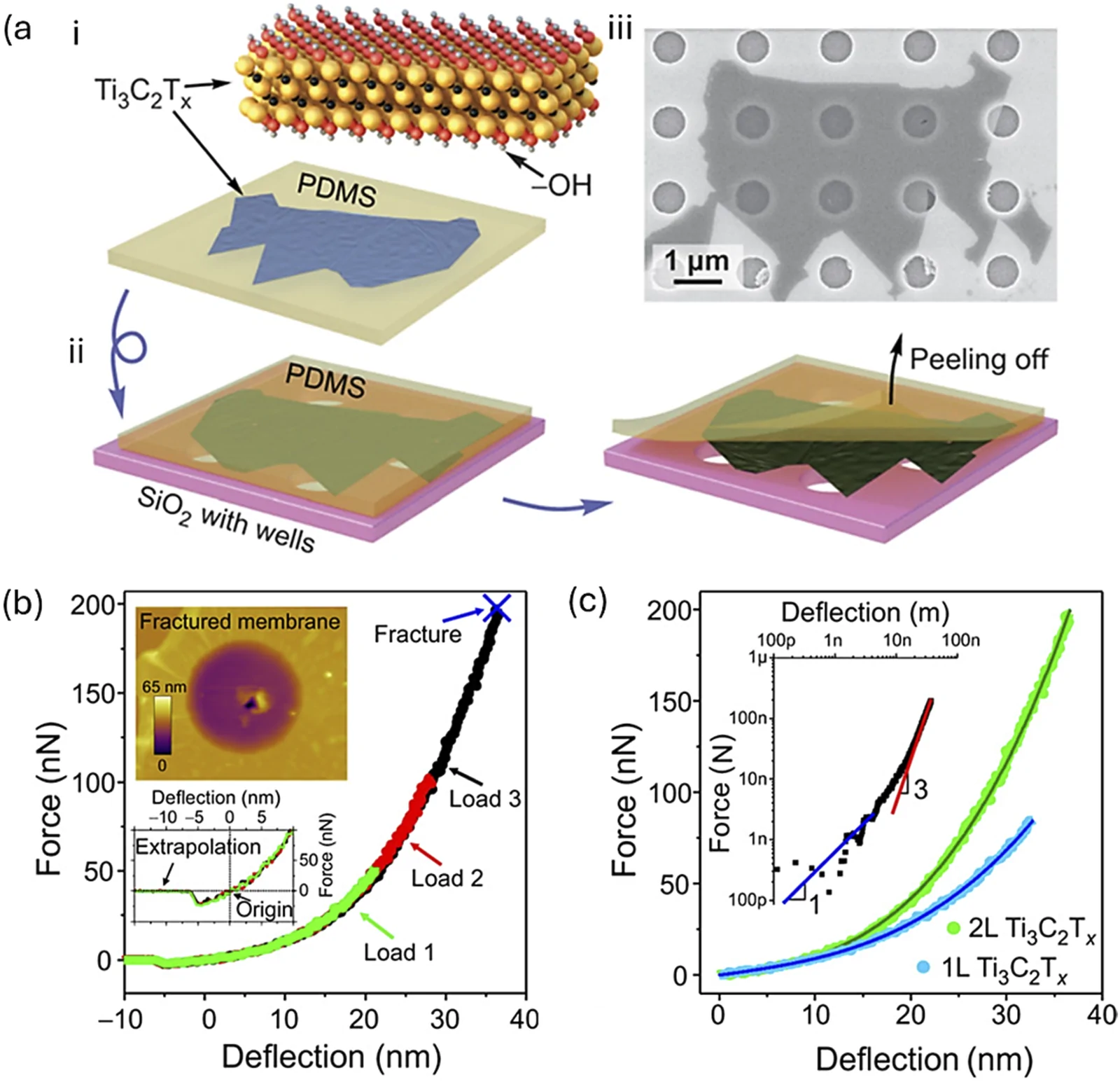
Preparation of MXenes (a(i)) Ti_3_C_2_T_*x*_ monolayer membranes (yellow spheres, Ti; black spheres, C; red spheres, O; gray spheres, H). (a(ii)) Scheme of the polydimethylsiloxane (PDMS)–assisted transfer of MXene flakes on a Si/SiO_2_ substrate with prefabricated microwells. (a(iii)) SEM image of a Ti_3_C_2_T_*x*_ flake covering an array of circular wells in an Si/SiO_2_ substrate with diameters of 0.82 µm.^55^ (b) Force-deflection curves of a bilayer Ti_3_C_2_T_*x*_ flake at different loads. The bottom inset is a detailed view of the same curves showing the center of origin. The top inset shows an AFM image of the fractured membrane.^55^ (c) Comparison of loading curves for monolayer (1L) and bilayer (2L) Ti_3_C_2_T_*x*_ membranes and the least squares fit to the experimental indentation curves (hole diameter is 820 nm). The curve shows a linear behavior in the first 10 nm of indentation (blue line) and approaches a cubic behavior at high loads (red line).^55^ This figure has been adapted/reproduced from ref. 55 with permission from the American Physical Society, copyright 2015.

The mechanical properties of MXene are determined by the specific combination of M–X components (M–C or M–N), surface terminators (M–O, M–OH, M–F or M–Cl), and the structure of MXene.^50–53^ The mechanical properties of two-dimensional titanium carbide were investigated in this study using classical molecular dynamics. Young's modulus was calculated from the linear portion of the stress–strain curves obtained during tensile deformation of the samples. Alexey *et al.* studied the mechanical properties of MXene through mechanical measurements of the elastic modulus and fracture toughness of monolayer and bilayer Ti_3_C_2_T_*x*_ MXene flakes using atomic force microscopy (AFM) indentation. We also compared MXene flakes with GO flakes, since both materials can be processed in solution due to their surface functionalization. The three atomic layers of Ti are packed in the stacking order, the octahedral sites are filled with carbon atoms, and the flakes are terminated with –F, –O or –OH groups (Fig. 4a(i)). For the preparation of MXene thin films, the MXene solution is directly poured onto the PDMS support and dried in air, leaving many MXene flakes on the surface.^54,55^ The hydrophilic MXene flakes will have a stronger attractive interaction with the hydrophilic silica surface than the hydrophobic PDMS, as shown in Fig. 4a(i) and (ii). An atomic force microscope (AFM) in tapping mode was used to scan for MXene flakes suspended over wells; the AFM tip was placed at the center of a selected well, then moved downward to control the elongation of the MXene flake. The load was increased to 50 nN, and loading and unloading cycles were performed, as shown in Fig. 4b. The extension and contraction curves during each loading cycle, as well as the curves for different loads, converge with each other, indicating the high elasticity of the MXene flakes, with no flake separation observed during the measurement.

The applicability of 
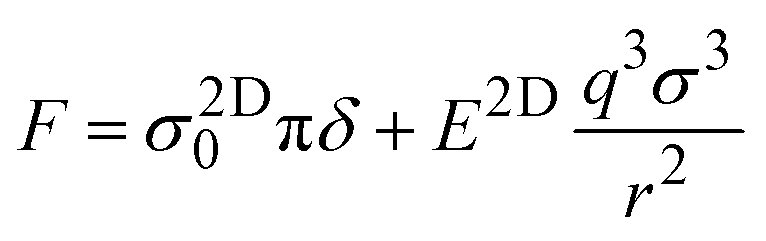
 is demonstrated in the inset in Fig. 4c, where the *F*–δ dependence for a bilayer Ti_3_C_2_T_*x*_ flake is shown on a logarithmic scale.^55^ A linear dependence is shown at small loads, while at loads larger than 10 nN a cubic dependence is observed. This indicates that the mechanical responses are characterized by a cubic relationship. Fig. 4c shows the experimental and fitted curves for single- and double-layer MXene flakes. A good fit (*R*^2^ > 0.995 for all measurements) is an indication of a good model fit.

### 3.3. Electronic properties

**Fig. 5:**
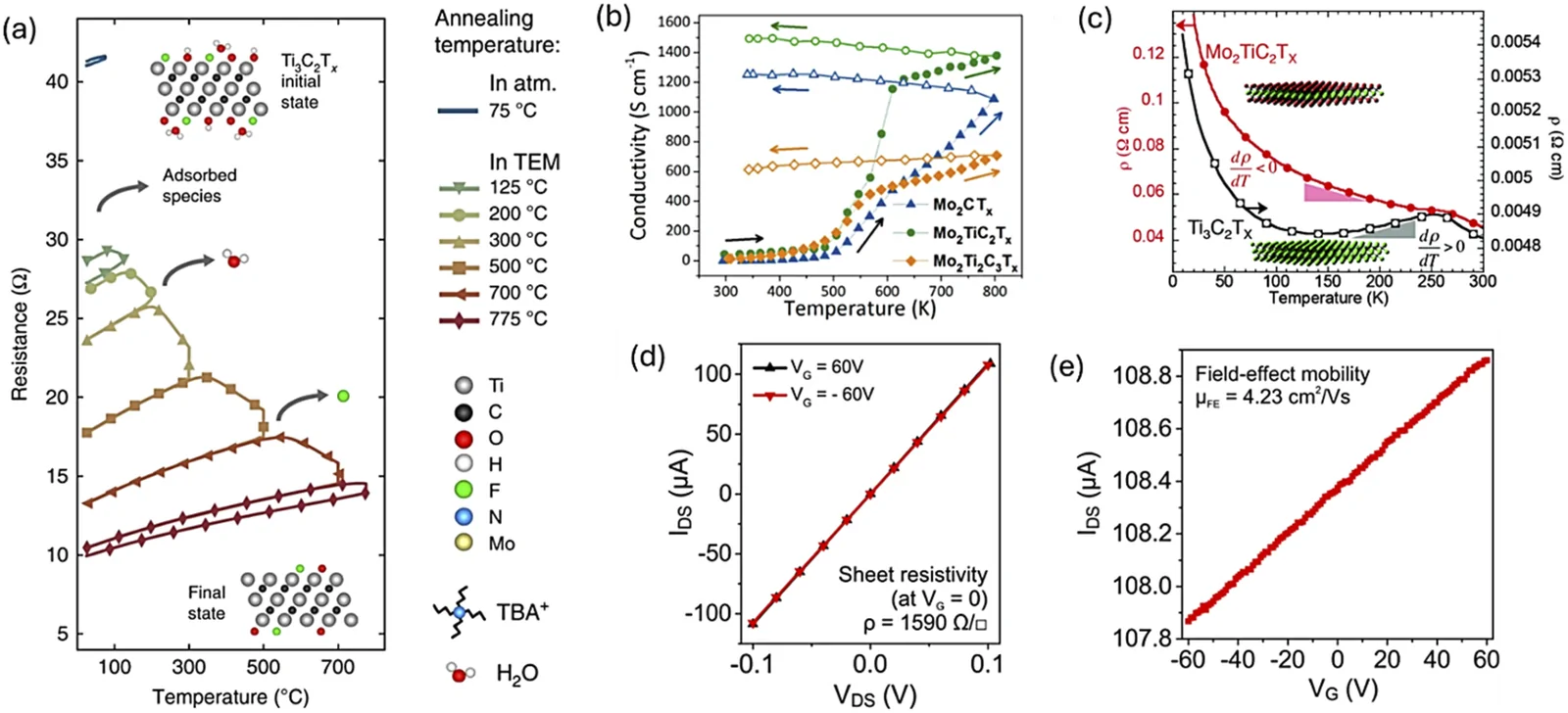
(a) Evolution of MXene electronic properties with *in situ* vacuum annealing. Resistance *versus* temperature measurements are shown for Ti_3_C_2_T_*x*_.^62^ Adapted from ref. 62, licensed under CC BY 4.0. (b) Electrical conductivity is affected by temperature-dependent thermoelectric properties of Mo-based MXenes.^71^ (c) Comparison of resistivity between Mo_2_TiC_2_T_*x*_ and Ti_3_C_2_T_*x*_.^72^ (d) *I*_DS_−*V*_DS_ curves for the device shown at different gate voltages.^73^ (e) *I*_DS_−*V*_G_ dependence of the device.^73^ This figure has been adapted/reproduced from ref. 71 with permission from Wiley-VCH, copyright 2018.

The 2D electronic properties strongly depend on the MXene synthesis method. Further characterization of individual Ti_3_C_2_T_*x*_ flakes will establish the importance of optimizing the synthesis conditions for Ti_3_C_2_T_*x*_, provide insights into its intrinsic properties, and reveal its potential for relevant applications. MXenes with good electrical conductivity properties can be modified by changing the composition, morphology, and terminal groups. The surface functionalities can significantly change the electronic band structure of MXene, leading to changes in free carrier density and effective mass.^56–60^ In addition, the electrical conductivity of MXene is also affected by surface termination points,^61–63^ temperature,^64^ ion intercalation,^65,66^ doping, pressure,^67^ grain boundaries, and strain.^68^ James L. *et al.* reported the control of MXene's electronic properties *via* termination and intercalation, and density functional theory (DFT) studies have consistently predicted that surface functionalization reduces the Ti_3_C_2_T_*x*_ density of states (DOS) at the Fermi level (*E*_F_), suggesting a decrease in the charge carrier density and thereby a decrease in the conductivity (Fig. 5a). Furthermore, high-temperature annealing and partial loss of surface terminations (Fig. 5a, bottom insets) increased the conductivity of MXene.^67^ This finding confirms that non-terminated MXene exhibits metallic behavior with high carrier concentrations.^69–71^ The Seebeck coefficient for the thermoelectric effect and conductivity can be modified by changing the elemental composition in molybdenum-based MXene, as in Mo_2_TiC_2_T_*x*_ MXene, which has a thermoelectric power of 3.09 × 10^−4^ W m^−1^ K^−2^ at 803 K.^71^Fig. 5c also provides a comparison of the resistivity between the Mo-containing MXenes and Ti_3_C_2_T_*x*_ samples, both of which show a change in behavior near 250 K. For Ti_3_C_2_T_*x*_ from 130 to 250 K it is metallic, in contrast to Mo_2_TiC_2_T_*x*_, where the resistivity increases with decreasing temperature over the measured range of 10 to 250 K. The resistivity of Ti_3_C_2_ increases with decreasing temperature below 130 K.^72^

Alexey *et al.* also studied the electrical properties of Ti_3_C_2_T_*x*_ by measuring the source-drain (IDS) current, applying a gate voltage (*V*_G_) to the final electrode. Fig. 5d shows a linear dependence, indicating ohmic behavior. The calculated sheet resistivity at *V*_G_ = 0 is *ρ* = 1590 Ω^−1^, which is comparable to the sheet resistivity of graphene, demonstrating good interlayer conductivity through the surface functional groups. In addition, the external electric field applied through the gate electrode shifts the Fermi level of the MXene flakes, leading to a change in the IDS. In Fig. 5e, IDS increases as *V*_G_ increases, indicating that electrons are major charge carriers, the electric field effect is intrinsic, and leakage current is negligible.^73^ On the other hand, small metal ion intercalations can enhance the particle density in the MXene sample, leading to increased conductivity with ions such as K and Na. The highest conductivity of MXene is obtained in the Li ion-intercalated Ti_3_C_2_T_*x*_ MXene, with a value of 9880 S cm^−1^.^66^ We know that the conductivity of MXenes is anisotropic,^67,68^ making the effective masses of electrons and holes depend on the direction; thus, the conductivity in the plane will be higher than the conductivity in a direction perpendicular to the basal plane.^68^

### 3.4. Optical properties

#### 3.4.1. Nonlinear optics

**Fig. 6:**
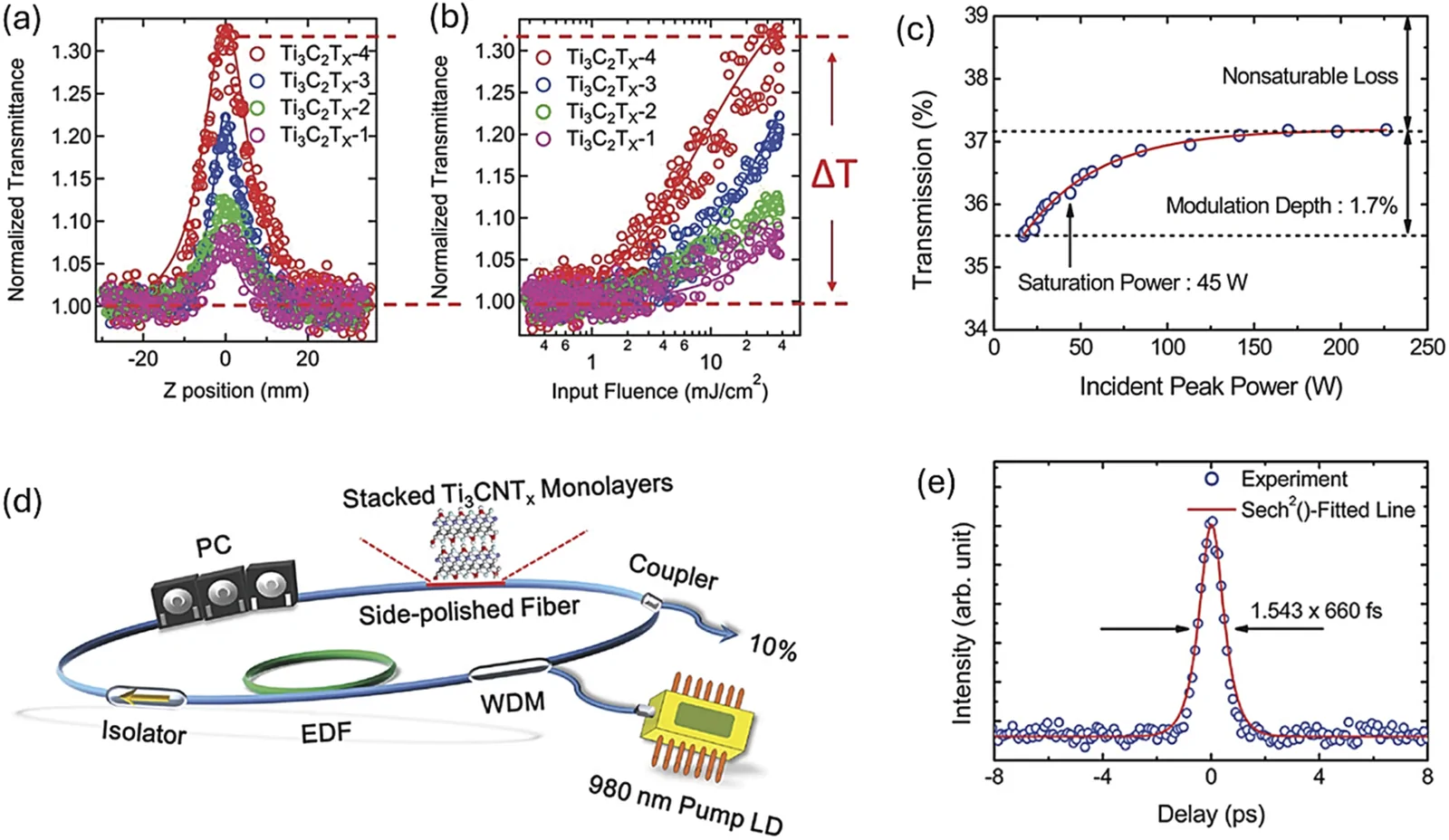
(a) Variation in nonlinear transmittance, or modulation depth (Δ*T*), of Ti_3_C_2_T_*x*_ films across different positions, characterized by the Z-scan method.^75^ (b) Intensity-dependent nonlinear transmission of Ti_3_C_2_T_*x*_ films shown as a function of position.^75^ (c) Nonlinear transmission curve as a function of energy for a side-polished fiber coated with a Ti_3_CNT_*x*_ monolayer.^78^ (d) Schematic of a ring-cavity erbium-doped fiber laser setup utilizing stacked Ti_3_CNT_*x*_ saturable absorbers (SAs). Abbreviations: PC–polarization controller, EDF–erbium-doped fiber, WDM–wavelength division multiplexer, LD–laser diode.^78^ (e) Measured autocorrelation trace.^78^ This figure has been adapted/reproduced from ref. 78 with permission from the American Physical Society, copyright 2014.

The linear optical properties of MXene have recently been demonstrated; these are related to light–matter interactions where the material responds nonlinearly to the electromagnetic field.^74,75^ The linear absorption losses are lower, and the light absorption of Ti_3_C_2_T_*x*_ tends to become nonlinear, which is known as saturable absorption, and this gives rise to the nonlinear behavior in MXene.^76,77^ As shown in Fig. 6a and b, the z-scan experiments measured a position-dependent change in the nonlinear transmittance, and the nonlinear transmission of Ti_3_C_2_T_*x*_ films for a current density of 40 mJ cm^−2^ was calculated and plotted as a function of intensity at each sample position.^75^ The effective nonlinear absorption coefficient decreases with increasing illumination intensity until a threshold intensity is reached. A nonlinear transmission curve (Fig. 6c) indicative of saturable absorption was clearly observed in this fiber-optic device under a picosecond pulsed laser input with a wavelength of 1560 nm and a repetition rate of 22 MHz. The saturation power and the modulation depth of MXene were estimated to be 45 W and 1.7%, respectively.^78^ Saturable absorption behavior was exhibited by all the Ti_3_C_2_T_*x*_ films with different thicknesses. Metallic Ti_3_C_2_T_*x*_ and Ti_3_CNT_*x*_ were used in femtosecond mode-locked (Fig. 6d).^78^ By using a two-photon absorption-based autocorrelator, it is shown that Ti_3_CNT_*x*_ SAs can be applied as ultrafast mode locks that can generate femtosecond laser pulses with the temporal width of the output pulses as short as 660 fs (Fig. 6e).^78^

#### 3.4.2. Plasmonic properties of Ti_3_C_2_T_*x*_ MXenes

Surface plasmons are formed by the interaction of light and matter at the metal surface and are a collection of free electrons at the metal–dielectric interface.^79^ Plasmonic materials, such as transition-metal nitrides and transparent conducting oxides, exhibit tunability and mechanical strength unlike noble metals. MXenes have become ideal plasmonic materials due to their metallic nature and chemical stability, and have found wide applications, for example, as optical sensors and in surface-enhanced Raman spectroscopy and optoelectronic devices.^80–82^ The dielectric properties of multilayer Ti_3_C_2_T_*x*_ were investigated by combining various spectroscopic techniques with *ab initio* calculations. In addition, the weaker interlayer interactions of MXene and the change of the functional groups in Ti_3_C_2_T_*x*_ also contribute to surface plasmon enhancement.^82^

Beyond these intrinsic properties, the plasmonic performance of MXene-based photonic devices is strongly governed by interface energetics. The work function of Ti_3_C_2_T_*x*_ is highly sensitive to surface terminations, oxidation, and interfacial chemistry, which directly affect energy-level alignment and charge-transfer efficiency at heterogeneous interfaces. Consequently, rational interface engineering is essential to promote hot-carrier injection, suppress carrier recombination, and enhance carrier transport in plasmon-enhanced photonic devices. For example, Xiangqian *et al.* developed an AgNP@MXene hybrid in which plasmonic Ag nanoparticles act as efficient photon harvesters while Ti_3_C_2_T_*x*_ provides highly conductive charge–transport pathways.^83^ The strong electronic coupling at the Ag/MXene interface facilitates localized surface plasmon resonance (LSPR)-induced hot-electron generation and rapid charge separation, resulting in enhanced photothermal conversion and photoelectric performance. These findings indicate that the superior performance of MXene-based plasmonic systems arises not only from the intrinsic optical properties of MXenes but also from optimized interfacial energetics and device architecture.

**Fig. 7:**
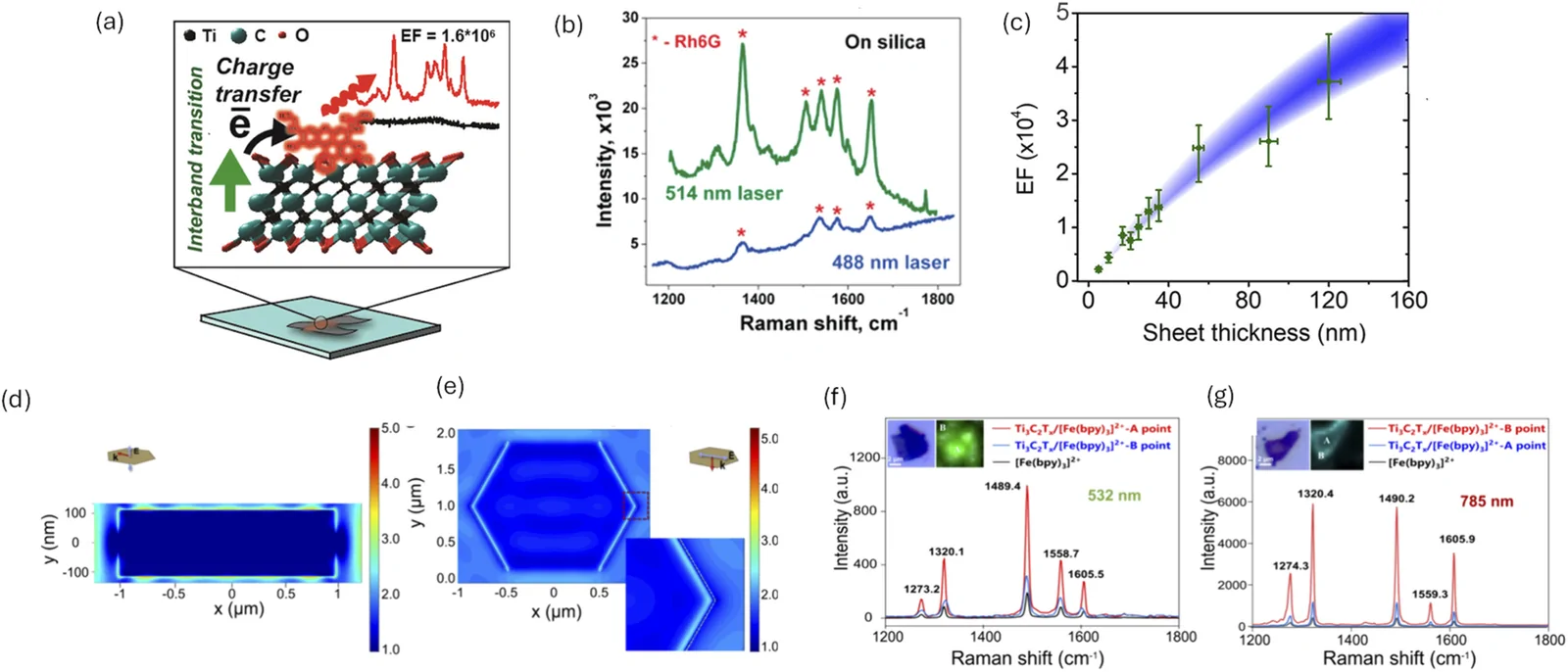
(a) Proposed mechanism for SERS, where field enhancement is attributed to hot spots (depicted in red as surface roughness). Interband transitions within MXene flakes contribute to strong polarization effects that promote SERS and facilitate enhanced charge transfer to the dye molecules.^89^ (b) Raman signal enhancement observed using 488 nm and 514 nm laser excitations on an MXene/SiO_2_/Si substrate.^89^ (c) Graph showing the relationship between Raman enhancement factor (EF) and the thickness of Ti_3_C_2_T_*x*_ sheets under 532 nm laser excitation.^90^ This figure has been adapted/reproduced from ref. 90 with permission from the American Chemical Society, copyright 2017. (d) FDTD-calculated electric-field enhancement distributions (|*E*|*E*_0_|) of a few-layer Ti_3_C_2_T_*x*_ MXene under incident light at 365 nm (d) and 850 nm (e), highlighting wavelength-dependent near-field localization. (f and g) Raman enhancement of [Fe(bpy)_3_]^2+^ on a few-layer Ti_3_C_2_T_*x*_ MXene measured under 532 nm (f) and 785 nm (g) laser excitations.^91^

Ti_3_C_2_T_*x*_ MXenes are also promising for surface-enhanced Raman scattering (SERS) applications due to their abundant surface terminators and photoelectric properties.^84–88^Fig. 7a shows the Raman signal enhancement mechanism at the hot spot due to the interband transition to the empty energy state of the functional groups of MXene, followed by charge transfer to the absorbed molecule; the photon emission relaxation process in the form of a Raman scattering signal is then recorded.^89^ Specifically, in Fig. 7b, to evaluate the dependence of SERS performance on Ti_3_C_2_T_*x*_ deposition quality, R6G molecular concentration was drop-cast on a Si/SiO_2_ substrate by Ti_3_C_2_T_*x*_ sputter coating. The results show a strong enhancement in the Raman signal, with enhancement factors of ∼1.2 × 10^6^ and 5.3 × 10^5^ for the 488 and 514 nm lasers, respectively.^89^ The dependence of the Raman enhancement factor on the thickness of the MXene sheets is also given in Fig. 7c. The monotonic increase of Raman EF for Methylene blue with increasing Ti_3_C_2_T_*X*_ sheet thickness was also probed with a 532 nm laser. Increasing the sheet thickness from 5 to 120 nm led to an increase in the enhancement factor from 2.2 × 10^3^ to 3.7 × 10^4^.^90^

The Raman enhancement observed on Ti_3_C_2_T_*x*_ MXenes arises from a synergistic combination of electromagnetic field concentration at surface roughness sites and chemical enhancement through photoinduced charge transfer. Upon optical excitation, interband transitions within Ti_3_C_2_T_*x*_ generate energetic carriers that can be transferred to the adsorbed Rh6G molecules, forming transient charge-transfer states that enhance molecular polarizability and Raman scattering efficiency. The significantly stronger Raman response under 514 nm excitation suggests a favorable resonance condition between the excitation energy and the charge–transfer transition. Furthermore, the monotonic increase in enhancement factor with MXene thickness indicates that thicker Ti_3_C_2_T_*x*_ sheets provide a higher density of electronic states, stronger optical absorption, and more efficient charge–transfer pathways, thereby amplifying the chemical contribution to SERS. This thickness-dependent behavior highlights the critical role of interfacial electronic coupling in determining the Raman enhancement performance of MXene-based substrates.

Wu *et al.* reported the anisotropic LSPR features of Ti_3_C_2_T_*x*_ MXenes as well as their site-selective photocatalysis enabled by the photophysical anisotropy.^91^ In particular, Fig. 7d–g demonstrate the spatially localized nature of plasmon-induced enhancement in Ti_3_C_2_T_*x*_ MXenes. Electromagnetic simulations reveal pronounced electric-field confinement at the edges and corners of the flakes, indicating anisotropic plasmon excitation and the formation of localized hot-carrier generation sites. The position-dependent Raman spectra of Fe(bpy)_3_^2+^ collected under 532 and 785 nm excitation further confirm the nonuniform enhancement behavior predicted by the simulations. The stronger Raman response observed at selected edge regions originates from efficient plasmon-assisted charge transfer between Ti_3_C_2_T_*x*_ and the adsorbed molecules, which increases molecular polarizability and Raman scattering efficiency. The enhanced response under 532 nm excitation suggests a more favorable resonance condition for interband-transition-mediated charge transfer, whereas the persistence of enhancement at 785 nm reflects the broadband plasmonic nature of Ti_3_C_2_T_*x*_. These results establish that anisotropic plasmon resonances in MXene enable spatially selective hot-carrier generation and site-specific molecular activation, providing a mechanistic basis for plasmon-driven catalysis on two-dimensional MXene surfaces.

#### 3.4.3. Ultrafast dynamics

**Fig. 8:**
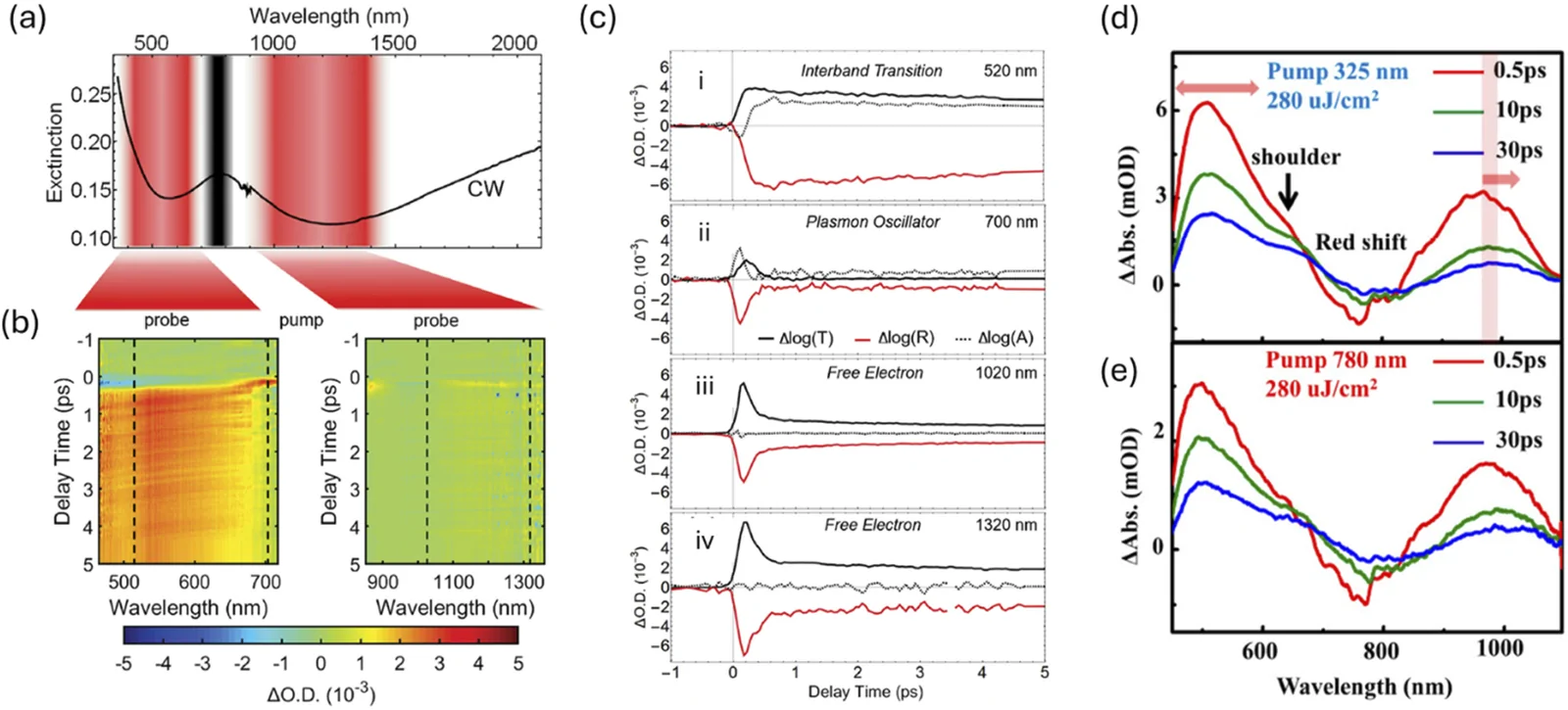
(a) A 785 nm pump pulse with 100 fs resolution initiates the process. An initial probe pulse either in the visible or infrared range is introduced, and both transmission and reflection are recorded simultaneously by separate detectors. The sample is then excited by the pump pulse, and a delayed probe pulse is used to measure the response over time.^93^ (b) Sapphire's response is removed from the transmission and reflection data to accurately calculate the time-dependent changes in absorption.^93^ (c) Transient data showing transmission (black), reflection (red), and the derived absorption (dashed black) for selected wavelengths. The spectra reveal how MXenes in solution respond to different excitation wavelengths and lattice temperatures, helping to analyze the thermal dynamics of MXene nanosheets.^93^ (d and e) Transient photoinduced absorption spectra captured at 0.5, 10, and 30 picoseconds under excitations of 325 nm and 780 nm, respectively, using a pump fluence of 280 µJ cm^−2^.^92^ This figure has been adapted/reproduced from ref. 92 with permission from the American Chemical Society, copyright 2021.

A deep understanding of the carrier dynamics in optically excited MXenes is an important factor for the effective use of MXenes in photonics and optoelectronic applications. Electron and phonon dynamics in MXenes occur in the femtosecond and picosecond ranges and can be analyzed using ultrafast spectroscopic techniques.^92–95^ Ultrafast pump-probe measurements of Ti_3_C_2_T_*x*_ MXene thin films were conducted, in which a broadband probe was impinged on the sample, and transmission and reflection were measured using two separate probes. The pump pulse illuminates the sample, exciting conduction and bound electrons. The excited electrons decay back to equilibrium on the nanosecond time scale. Electrons excited at a time delay with respect to the pump pulse are measured by a second broadband probe pulse (Fig. 8a).^93^ The results show the pump-induced change in the optical response at a time resolution of 100 fs. Fig. 8b also gives a transient absorption profile from approximately 450–720 nm and 860–1350 nm for time values from −1 to 5 ps, as indicated by the black dashed line.^93^

The simultaneous ultrafast transmission and reflection (SUTR) response was calibrated from both transmission and reflection measurements of the Ti_3_C_2_T_*x*_ film, with transient events selected at wavelengths of 520, 700, 1020 and 1320 nm (Fig. 8c). Depending on the probe wavelength, the dynamics can be attributed to interband transitions (the interband response for wavelengths shorter than 600 nm) and the 800 nm SP resonance (dominated by the free electron response for wavelengths longer than 1000 nm). The pump-induced reflection artifact gradually decreases after the pump pulse, and the pump-induced transmission changes are positive for all cases.^93^Fig. 8d and e shows the transient absorption spectra for MXene in solution at different time stages under photoexcitation at 325 nm and 780 nm. The transient absorption spectra all show photoinduced absorption enhancement in the visible region from 450 nm to 680 nm and the near-infrared region from 850 nm to 1100 nm at 0.5 ps.^94^ Ultrafast kinetics is a phase that strongly depends on the excitation and probe wavelengths. The fundamental ultrafast process in Ti_3_C_2_T_*x*_ MXenes has been deeply and clearly investigated through ultrafast kinetics studies over a wide range of pump and probe wavelengths. This provides an important step towards the use of Ti_3_C_2_T_*x*_ MXenes in photothermal and nano-optical devices.

## 4. Ti_3_C_2_T_*x*_ MXenes for photonic applications

Ti_3_C_2_T_*x*_ MXenes have optoelectronic properties as well as superior mechanical strength, conductivity and surface functionality, making them highly promising candidates for many applications in advanced optoelectronic devices. The light absorption ability and high photodetection potential of MXenes have important roles in photodetectors, lasers and light-emitting devices in integrated optoelectronics.^96–100^

The practical implementation of MXenes in photonic devices is critically governed by their environmental stability, as these materials are highly susceptible to oxidation under ambient conditions. Oxidative degradation typically initiates at flake edges and defect sites, where exposure to oxygen and moisture leads to the gradual transformation of the metallic carbide lattice into corresponding oxides, accompanied by structural fragmentation and loss of electrical conductivity. This process is further accelerated by light irradiation and thermal effects during device operation, resulting in diminished optical absorption, suppressed nonlinear response, and overall performance degradation.^101–104^ To mitigate these effects, careful storage protocols, such as low-temperature conditions, inert atmospheres, and the use of deoxygenated solvents, are commonly employed to retard oxidation kinetics. More importantly, encapsulation strategies play a pivotal role in extending the functional lifetime of MXene-based photonic systems. Hybrid encapsulation approaches that combine organic and inorganic layers have demonstrated enhanced barrier performance and improved device stability. Despite these advances, the operational lifetime of MXene-integrated photonic devices remains sensitive to environmental exposure, optical power density, and electrical bias, with unprotected systems degrading within hours to days, whereas properly encapsulated devices can maintain performance over extended periods. Consequently, the development of robust surface chemistries, advanced encapsulation architectures, and optimized operating conditions is essential for enabling reliable and durable MXene-based photonic technologies.^103–105^

### 4.1. Nonlinear photonic devices

**Fig. 9:**
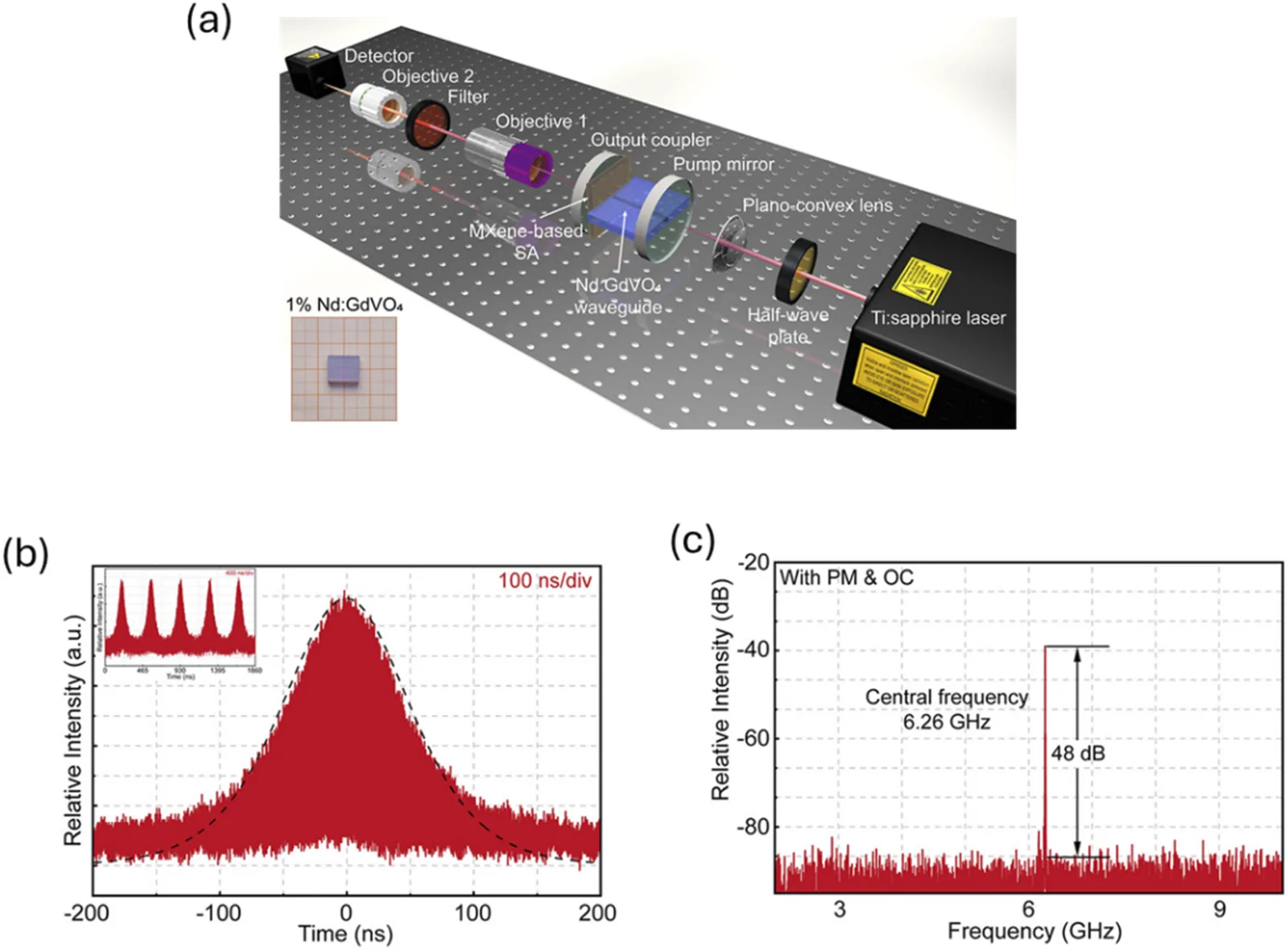
(a) Diagram of a standard end-face coupling setup used for evaluating guiding properties and generating Q-switched mode-locked (QML) laser pulses. The inset shows an optical image of the Nd : GdVO_4_ laser crystal.^106^ (b and c) Single Q-switched envelope is shown, with an inset displaying the pulse train, and the corresponding RF spectrum of the QML laser under conditions involving a polarization modulator (PM) and an output coupler (OC).^106^ This figure has been adapted/reproduced from ref. 109 with permission from Wiley-VCH, copyright 2019.

Ti_3_C_2_T_*x*_ is one of the studied MXene materials with electrical conductivity and optical transmission covering the spectral region from ultraviolet (UV) to mid-infrared (MIR). Ti_3_C_2_T_*x*_ materials have suitable optoelectronic properties and exhibit enhanced light–matter interaction as well as an ultrafast nonlinear optical response. Li *et al.* presented a study on the optical modulation capability of 2D MXene for ultrafast photonics applications by incorporating SA Ti_3_C_2_T_*x*_ and Ta_4_C_3_T_*x*_ with waveguide structures.^106^Fig. 9a shows the typical end-face coupling system for guiding property characterization and Q-switched mode-locked laser generated by Ti_3_C_2_T_*x*_. The Q-switched path with a full width at half maximum (FWHM) of 103 ns is formed by the mode-locked sequence, and the inset depicts the Q-switched pulse trains on the nanosecond time scale (400 ns div^−1^), as shown in Fig. 9b. Fig. 11c shows the radio frequency (RF) spectrum, and a sharp and clear peak can be observed at 6.26 GHz with a signal-to-noise ratio (SNR) of 48 dB. The waveguide laser contains a higher cavity power density and enhanced gain compared to other lasers, resulting in powerful Q-Switched Mode-Locked laser emission.^106^

In nonlinear applications such as saturable absorbers (SAs) for mode-locking or optical switching, MXenes are limited by their low laser damage threshold and photothermal sensitivity. While their high nonlinear absorption coefficient and ultra-fast carrier dynamics are advantageous, MXenes possess an inherent high photothermal conversion efficiency. Under the intense fluences required for nonlinear effects, localized heat accumulation often exceeds the material's structural tolerance, leading to the irreversible oxidation of the MXene flakes into insulating oxides. This phase transition not only destroys the nonlinear response but also increases the non-saturable loss within the optical cavity. Furthermore, the scattering loss induced by the random orientation and polydispersity of MXene flakes in solution-processed films can significantly degrade the *Q*-factor of photonic resonators, complicating the achievement of high-performance, low-threshold nonlinear modulation. Table 1 summarizes the detailed characteristics of ultrafast lasers enabled by different MXene-based saturable absorbers. According to DFT calculations, MXene-based saturable absorbers possess tunable bandgap energies and a broadband optical response, which make them promising candidates for ultrafast pulsed lasers.

**Table 1:** Characteristics of ultrafast lasers enabled by different MXene-based saturable absorbers

Materials	Laser type	λ [nm]	SNR [dB]/τ [s]	Energy [mW/J]	*f*_rep_	Ref.
Ti_3_C_2_T_*x*_ MXene	*Q*-switched Nd : YAG ceramic laser	1064	—/359 ns	94.8 mW/0.66 µJ	186 kHz	107
V_2_CT_*x*_ MXene	Er-doped fiber laser	1559.1	55 dB/3.21 ps	—	4.9 MHz	108
Ti_3_C_2_T_*x*_ MXene	Er-doped fiber laser	1566.9 nm	36 dB/850 fs	6.95 Mw/0.032 nJ	218.4 MHz	109
α-Mo_2_C MXene	Er-doped or Yb-doped fiber laser	1602.6–1061.8 nm	64 dB/0.48 ps	127.1 mW/80 µJ	1.88 MHz	110
Ti_3_CN MXene	Er-doped fiber laser	1557 nm	60 dB/660 fs	0.05 mW	15.4 MHz	111

Ti_3_C_2_T_*x*_ MXenes have emerged as one of the most promising two-dimensional materials for ultrafast photonic applications owing to their broadband optical absorption, metallic conductivity, and ultrafast carrier dynamics. As summarized in Table 2, Ti_3_C_2_T_*x*_-based saturable absorbers exhibit broadband operation spanning from the visible and near-infrared regions (600–800 nm) to the mid-infrared regime (1900 nm), demonstrating remarkable wavelength versatility for passive Q-switching and mode-locking applications. The reported saturation fluence or saturation intensity varies considerably with material morphology and device architecture, ranging from several tens of mW cm^−2^ for evanescent-field fiber configurations to several GW cm^−2^ for quantum dots and thin-film devices under free-space excitation. In particular, microfiber and fiber-integrated Ti_3_C_2_T_*x*_ devices exhibit relatively low saturation thresholds (10–50 mW cm^−2^) and insertion losses below 2 dB, enabling efficient nonlinear modulation with reduced cavity losses. The carrier recovery dynamics of Ti_3_C_2_T_*x*_ are typically in the picosecond regime (1–6 ps), which originate from rapid electron–electron and electron-phonon scattering processes and support the generation of ultrashort optical pulses. By contrast, Ti_3_C_2_T_*x*_ quantum dots display longer recovery times on the nanosecond timescale, likely due to enhanced carrier localization and surface-mediated recombination pathways associated with quantum confinement effects. Furthermore, insertion losses generally remain below 5 dB for most device configurations, highlighting the favorable balance between nonlinear optical modulation and cavity efficiency. Collectively, these characteristics position Ti_3_C_2_T_*x*_ MXenes as highly competitive saturable absorber materials for broadband ultrafast photonics, although challenges associated with oxidation stability, surface termination control, and long-term environmental durability remain as important concerns for practical device deployment.

**Table 2:** Representative nonlinear optical parameters of Ti_3_C_2_T_*x*_ MXene materials for ultrafast photonic applications

Material	Wavelength range	Saturation fluence	Recovery time	Insertion loss	Ref.
Ti_3_C_2_T_*x*_ thin film	800 nm	70 GW cm^−2^	2 ps	N/A	112
Ti_3_C_2_T_*x*_ QD	1030–1064 nm	25–100 mW cm^−2^	1 ps	3 dB	113
Ti_3_C_2_T_*x*_	1060 nm	0.49 mJ cm^−2^	1 ps	2 dB	114
Ti_3_C_2_T_*x*_ film SA	1064 nm	0.7–1.2 mJ cm^−2^	1 ps	2 dB	115
Ti_3_C_2_T_*x*_ on microfiber	1550 nm	10–40 mW cm^−2^	2 ps	1.39 dB	116
Ti_3_C_2_T_*x*_ on side-polished fiber	1550 nm	10–50 mW cm^−2^	<1 ps	2 dB	117
Ti_3_C_2_T_*x*_	1900 nm	5–30 mW cm^−2^	<2 ps	5 dB	118
Ti_3_C_2_T_*x*_ nanosheets	800–1060 nm	150 mW cm^−2^	2–6 ps	2–6 dB	119,120
Ti_3_C_2_T_*x*_ nanosheets	1060–1550 nm	10–70 mW cm^−2^	3–5 ps	1.2–4 dB	121
Ti_3_C_2_T_*x*_ QD	600–1600 nm	1–2.5 GW cm^−2^	4–6 ns	0.7–2 dB	119

**Fig. 10:**
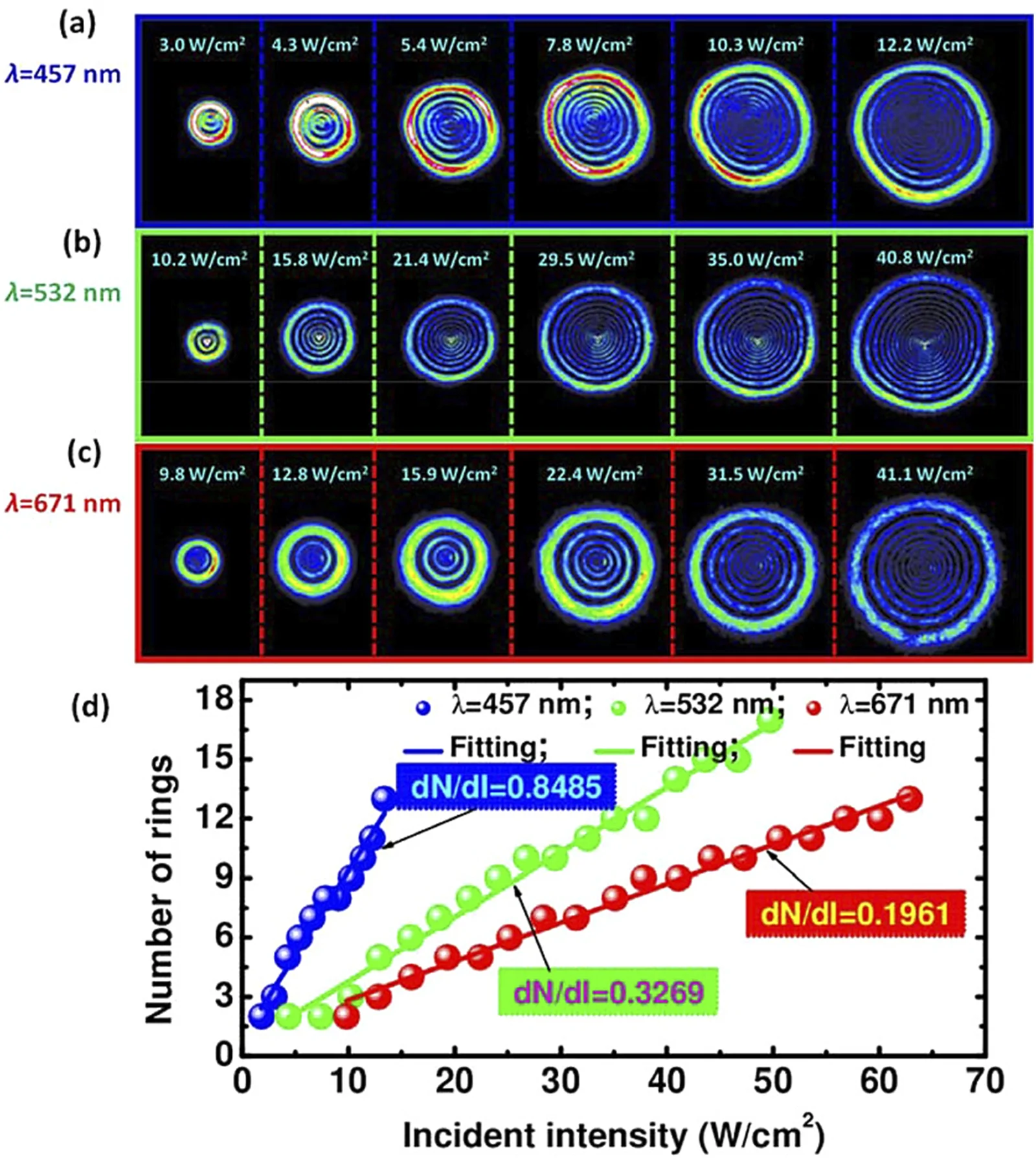
(a–c) Examination of diffraction rings and (d) their variations when utilizing the MXene charge-coupled device under different incident light conditions.^124^ This figure has been adapted/reproduced from ref. 121 with permission from Wiley-VCH, copyright 2019.

Moreover, Ti_3_C_2_T_*x*_ MXenes have been demonstrated to be effective saturable absorbers in stretched-pulse mode-locked fiber lasers. The resulting pulse train exhibits promising performance, with temporal widths of approximately 50.37 ns and 104 fs. Notably, no external electronic signal is required, as the parameters of one light beam can be modulated by another.^122,123^ For example, Wu and co-workers reported the nonlinear optical response of MXene using the self-scanning pump-probe technique.^124^ Upon evaluation, its performance surpasses that of conventional materials, such as Bi_2_Se_3_, graphene, and MoS_2_, exhibiting a nonlinear refractive index of approximately *n*_2_ ∼ 10^−4^ (Fig. 10). These findings indicate that the pump light can effectively modulate the nonlinear phase of the probe light due to the Kerr effect. The enhanced response is primarily attributed to the alignment and reorientation of Ti_3_C_2_T_*x*_ layers under the influence of the electromagnetic field. Yufeng *et al.* developed an all-optical device by coating MXene onto a microfiber operating in the telecommunication band. The optimized device exhibited a strong nonlinear optical response, with a conversion efficiency of −59 dB achieved at a signal frequency of 10 GHz.^125^

**Fig. 11:**
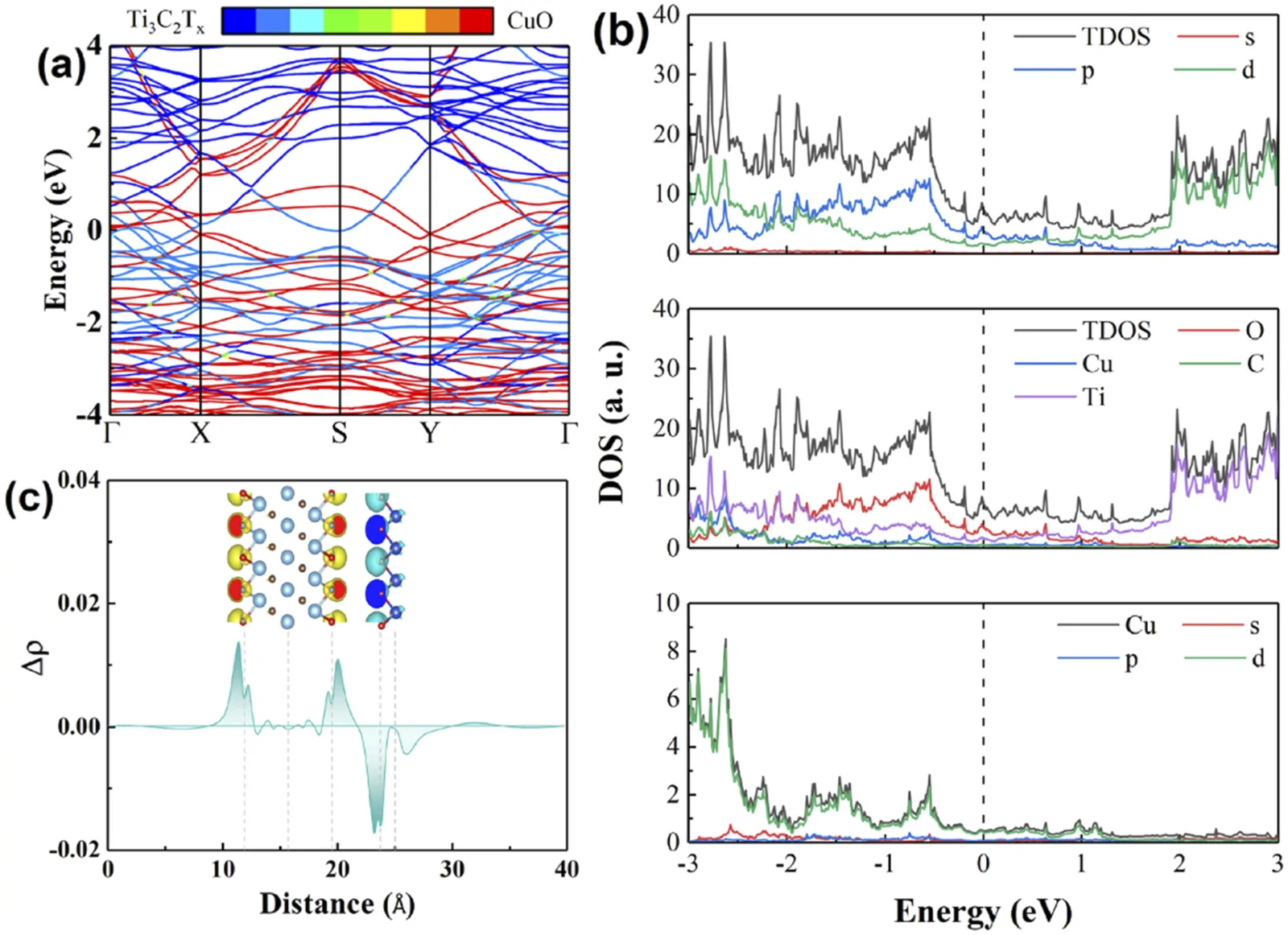
(a and b) Electronic band structure and density of states and the (c) planar-averaged charge density difference of the Ti_3_C_2_T_*x*_/CuO composite.^126^ This figure has been adapted/reproduced from ref. 115 with permission from Elsevier, copyright 2025.

Lihui and co-workers reported that Ti_3_C_2_T_*x*_/CuO composites exhibit promising performance as saturable absorbers in ultrafast lasers, with a pulse duration of approximately 495 fs and a SNR of 82.44 dB.^126^ A new band structure of the composites was investigated using DFT, which revealed band overlap that supports substantial carrier transition between the MXene and CuO. The density of states indicates that the d-orbitals play a dominant role in chemical bonding, particularly below the Fermi level, thereby regulating the electronic properties (Fig. 11). A built-in electric field is formed at the CuO/MXene interface, where electron depletion and accumulation are indicated by blue and yellow regions in Fig. 11c, respectively. This charge redistribution facilitates enhanced functionality in ultrafast photonic applications.

Representative studies, summarized in Table 3, demonstrate that broadband and near-perfect optical absorption in MXene-based photonic devices is primarily achieved through rational architectural design rather than the intrinsic optical properties of MXenes alone. Various device configurations, including metasurfaces, multilayer cavities, and refractory-metal resonator structures, employ localized surface plasmon resonances, Fabry–Pérot interference, impedance matching, and electric-field confinement to maximize light trapping and minimize reflection. Notably, the Ti_3_C_2_T_*x*_/W resonator architecture achieves an average absorptivity exceeding 95% across the 300–2500 nm solar spectrum, whereas other MXene-based metasurface absorbers typically exhibit absorption above 80–90% over broad wavelength ranges.^129^ These benchmarked examples highlight that careful structural engineering is the primary factor governing near-perfect absorption, while MXenes provide the favorable optical and electrical properties required for high-performance photonic devices.

**Table 3:** Representative MXene-based absorber architectures demonstrating broadband and near-perfect absorption for photonic applications

Device architecture	Active materials	Spectral range	Absorption performance	Dominant absorption mechanism	Key features/applications	Ref.
Three-layer metamaterial absorber with periodic square-ring nanocylinder metasurface	MXene/dielectric/metal ground plane	400–2400 nm	∼90% average absorption	LSPR	Polarization-insensitive; stable up to 60° incidence; suitable for solar cells, energy harvesting, and thermal imaging	127
Metamaterial absorber with periodic MXene nanorod metasurface	MXene	1150–2500 nm	>80% absorption	LSPR and Fabry–Pérot interference	Polarization-insensitive; wide-angle performance; suitable for infrared imaging and sensors	128
MXene/refractory-metal resonator absorber	Ti_3_C_2_T_*x*_/W	300–2500 nm	>95% average absorption (96.7% under AM1.5)	Hybrid resonances, impedance matching, and electric-field confinement	Near-perfect broadband solar absorber; solar energy conversion and photovoltaics	129
Double-multilayer metasurface absorber	MXene–SiO_2_–Fe	Visible–IR solar spectrum	>90% absorption	Metamaterial resonance and interference effects	Polarization-insensitive; efficient thermal harvesting and solar absorption	130

### 4.2. Ti_3_C_2_T_*x*_ MXene for photodetectors, solar cells, and light-emitting diodes (LEDs)

**Fig. 12:**
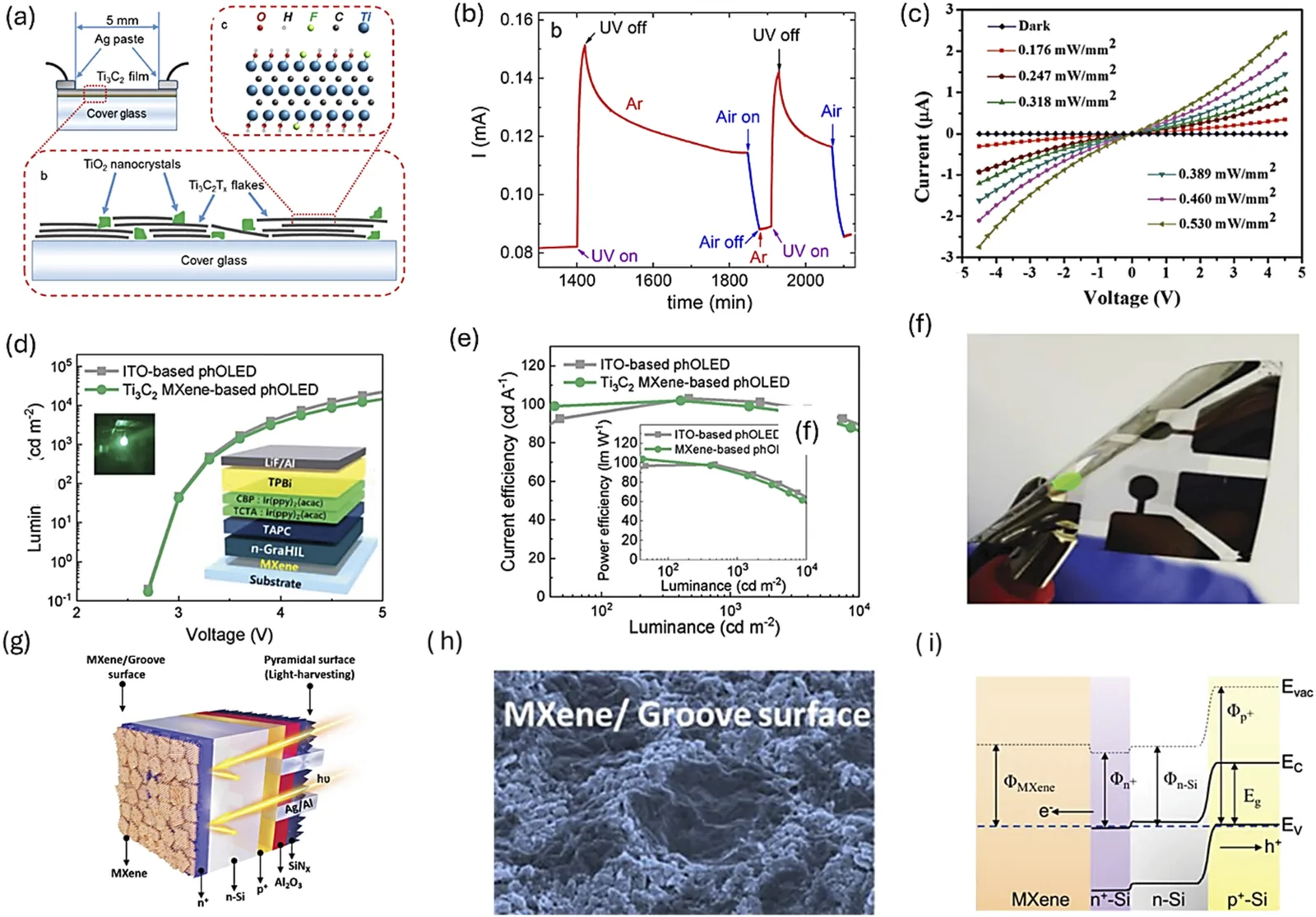
(a) Schematic of a Ti_3_C_2_T_*x*_ thin film on a cover glass with silver contacts, and a cross-sectional view highlighting the distribution of MXene flakes and TiO_2_ nanoparticles (in green) within and atop the MXene layers. (b) Time-dependent photoresponse of a 38 nm Ti_3_C_2_T_*x*_ film under UV light exposure and in ambient air over 30 minutes.^134^ (c) Current–voltage (*I*–*V*) curves of single photodetectors (PDs) both in the dark and under 405 nm light illumination at intensities from 0.176 to 0.53 mW mm^−2^.^133^ (d) Luminance *versus* voltage performance of LEDs using ITO or MXene electrodes; inset includes an optical image of a green MXene-based OLED (left) and a schematic of the green phosphorescent OLED structure (right).^151^ (e) Current efficiency *versus* luminance plot, with the inset showing the power efficiency *versus* luminance plot. (f) Photograph of flexible OLEDs employing MXene electrodes. (g) Structural diagram of a Ti_3_C_2_T_*x*_ MXene/n^+^np^+^-Si solar cell. (h) FE-SEM image of the MXene layer deposited on the n^+^ side of the silicon solar cell. (i) Proposed energy band diagram for the Ti_3_C_2_T_*x*_ MXene/n^+^np^+^-Si solar cell based on measured work function values, showing work function (*Φ*), silicon bandgap (*E*_g_), conduction band (*E*_c_), and valence band (*E*_v_).^152^ This figure has been adapted/reproduced from ref. 152 with permission from Wiley-VCH, copyright 2019.

Ti_3_C_2_T_*x*_ MXenes possess high electrical conductivity, broadband optical absorption, hydrophilic surfaces, and excellent mechanical flexibility, making them promising materials for photodetector applications.^131–133^ The performance of MXene-based photodetectors is governed by the synergistic interplay between plasmonic enhancement and interfacial charge–transfer processes. LSPR enhances the electromagnetic field in the vicinity of the photoactive layer, thereby increasing light absorption and promoting the generation of photocarriers. However, the enhanced photoresponse is not solely determined by plasmonic light harvesting. Efficient extraction and transport of photogenerated carriers require favorable energy-level alignment and strong electronic coupling at the MXene/semiconductor interface. Therefore, interface energetics, including work-function matching and interfacial charge transfer, are equally critical for suppressing carrier recombination and improving photodetector performance. Collectively, recent studies indicate that optimizing both plasmonic nanostructures and interface engineering is essential for achieving high-performance MXene-based photodetectors. As shown for the Ti_3_C_2_T_*X*_-TiO_2_-based photodetector depicted in Fig. 12a, the light response of Ti_3_C_2_T_*X*_-TiO_2_ was attributed to the electron transitions in TiO_2_ induced by UV irradiation. Fig. 12b shows the photoresponse of the Ti_3_C_2_T_*x*_ thin film with and without UV irradiation and exposure to ambient air for 30 min. After UV irradiation under an argon atmosphere, a rapid increase in current through the sample was observed with a subsequent slow decay, and upon transfer to ambient air, the photocurrent relaxation time resulted in a rapid decrease in the current. This suggests that the photoresponse of the Ti_3_C_2_T_*x*_ thin film is dependent on ambient conditions and could be applied to sensing devices.^134^ Wen *et al.* studied the photoelectric conversion efficiency of flexible photodetectors based on 2D CsPbBr_3_ nanosheets and a conductive Ti_3_C_2_T_*x*_ MXene electrode. Fig. 12c presents the I–V curves under 405 nm excitation light of different intensities. The photocurrent increases gradually with the increase in light intensity and tends to be nonlinear due to the influence of Schottky formation.^133^ In another study, the optimized InSe/MXene photodetector exhibits a fast response time of less than 2 ms, a high photocurrent of approximately 1 µA, a low dark current of around 5 nA, and a high detectivity of 7.1 × 10^11^ Jones under blue-light irradiation.^135^ To understand the role of MXene-based composites in photodetection applications, Koushik *et al.* performed DFT calculations to analyze the electronic band structure of Ti_3_C_2_T_*x*_/Bi_2_S_3_ hierarchical structures (Fig. S1).^136^ The DFT results show that the composite exhibits overlapping bands, indicating metallic behavior. Additionally, the total density of states (DOS) is shifted toward the Fermi level compared to pristine Bi_2_S_3_, suggesting enhanced electrical conductivity. The charge density difference plot (Fig. S1g) reveals regions of charge accumulation (yellow) and charge depletion (blue), confirming that electrons transfer from Bi_2_S_3_ to the Ti_3_C_2_T_*x*_ MXene *via* Bi–O bonds. This interfacial charge transfer between Ti_3_C_2_T_*x*_ and Bi_2_S_3_ plays a crucial role in facilitating efficient charge separation and transport, which is fundamental to achieving ultra-broadband photodetection performance. Table 4 presents a comparison of the performances of similar types of MXene-based photodetectors.

**Table 4:** Comparison of the performances of similar types of photodetectors

Material	Rise time	Delay time	Responsivity (*R*)	Ref.
Ti_3_C_2_T_*x*_/CsPbBr_3_	18 ms	46 ms	44.9 mA W^−1^	137
ZnO/Ti_3_C_2_T_*x*_	2.2 µs	6.8 µs	5.05 A W^−1^	138
Ti_3_C_2_T_*x*_/TiO_2_	45 ms	69 ms	2.06 mA W^−1^	139
Ti_3_C_2_T_*x*_/n-Ge	1.4 µs	4.1 µs	3.14 A W^−1^	140
MXene/Bi_2_Se_3_	19.7 µs	35.2 µs	24.7 mA W^−1^	141
2D Ti_3_C_2_-MXene nanosheets/ZnO	12.2 µs	16.1 µs	142.2 mA W^−1^	142
SnO_2_/MXene	63 ms	123 ms	64 mA W^−1^	143
Ti_3_C_2_T_*x*_/CdS	7.2 µs	120 µs	34.42 A W^−1^	144
Ti_3_C_2_T_*x*_/GO/GaAs	47.9 µs	45.9 µs	65 mA W^−1^	145
MXene/AlO_*x*_/Ge	73.5 µs	81.5 µs	665 mA W^−1^	146
MXene/h-BN/Si	55 µs	686 µs	3940 mA W^−1^	147
MXene/SiO_*x*_/Si	1.14 ms	1.6 ms	402 mA W^−1^	148
Te/MXene	12.2 ms	26.5 ms	1.84 A W^−1^	149
MXene–Cs_3_Bi_2_I_9_	0.27 ms	2.32 ms	6.45 A W^−1^	150

Blue-emitting organic LEDs were fabricated from solution-processed Ti_3_C_2_T_*x*_ MXene films as TCE. The high brightness and current density at a given applied bias are shown in Fig. 12d. The calculated relevant parameters of maximum power efficiency, external quantum efficiency, and maximum current efficiency show that MXene provides efficient hole injection into the EML of the LED (Fig. 12e). The LEDs fabricated with MXene anodes also show mechanical flexibility, green emission, and flexibility with PET/MXene anodes (Fig. 12f).^151^ A drop-cast Ti_3_C_2_T_*x*_ MXene is uniformly coated on the n^+^-Si macro-groove surface (Fig. 12h). The Ti_3_C_2_T_*x*_ MXene device contacts the rapid thermal annealing (RTA)-treated Si in an inert environment to improve the electrical contact between the MXene layer and n^+^np^+^-Si, enhance charge carrier transport, and reduce the contact resistance. Fig. 12g shows a schematic of Ti_3_C_2_T_*x*_ MXene-contacted Si solar cells. The band diagram of the Ti_3_C_2_T_*x*_ MXene/n^+^np^+^-Si solar cell is shown in Fig. 12i. The work function of the Ti_3_C_2_T_*x*_ MXene is higher than that of n^+^-Si and is enough to form an ohmic contact at the Ti_3_C_2_T_*x*_ MXene/n^+^-Si interface.^152^ This has led to an enhanced PCE of up to ≈11.5% with a 20% increase in the fill factor. Table 5 presents a summary of recent studies employing Ti_3_C_2_T_*x*_ MXenes as additives to improve the performance of perovskite solar cells.

**Table 5:** Summary of Ti_3_C_2_T_*x*_ MXene additives for enhanced perovskite solar cell performance

Device structure	*V*_oc_ (V)	*J*_sc_ (mA cm^−2^)	FF (%)	PCE (%)	Ref.
FTO/SnO_2_/CsFAMA-MXene(F)/Spiro-OMeTAD/Au	1.181	23.50	77.59	21.52	154
FTO/SnO_2_/CsFAMA-MXene(OH)/Spiro-OMeTAD/Au	1.175	23.38	75.83	20.83	
ITO/SnO_2_/MAPbI_3_-Ti_3_C_2_T_*x*_/Spiro-OMeTAD/Au	1.03	22.26	76.00	17.41	155
FTO/TiO2/CsPbI3/Ti_3_C_2_F_*x*_/Spiro-OMeTAD/Au	1.22	20.59	81.55	20.44	156
ITO/PTAA/CsPbI_3_/O-Ti_3_C_2_T_*x*_-CsPbI_3_/CPTA/BCP/Ag	1.21	19.86	81.96	19.69	157
ITO/NiO/MAPbI_3_ + Ti_3_C_2_T_*x*_/PCBM + Ti_3_C_2_T_*x*_/BCP/Ag	1.09	22.88	77.00	19.2	158
ITO/SnO_2_/(BA)_2_(MA)_4_Pb_5_I_16_-Ti_3_C_2_T_*x*_/Spiro-OMeTAD/Ag	1.11	20.87	67.84	15.71	159
ITO/SnO_2_/FA_1−*x*_MA_*x*_PbI_3−*y*_Br_*y*_ : Cs-Ti_3_C_2_T_*x*_/Spiro-OMeTAD/Au	1.10	26.00	76.00	21.57	160
ITO/SnO_2_/FA_1−*x*_MA_*x*_PbI_3−*y*_Br_*y*_ : p-Ti_3_C_2_T_*x*_/Spiro-OMeTAD/Au	1.10	24.12	75.00	18.89	

First-principles calculations typically report a broad and tunable work-function window by assuming uniformly terminated surfaces (–O, –OH, or –F terminations). In these idealized cases, the work function is governed by surface dipoles induced by specific functional groups, where O terminations generally yield higher work functions, while OH and F terminations lead to lower values due to differences in electronegativity and dipole orientation. However, experimentally synthesized MXenes rarely exhibit such uniformity; they possess mixed and dynamically evolving surface terminations, along with structural defects, adsorbates, and possible oxidation products. This heterogeneity leads to spatially averaged and often less predictable work-function values, typically within a narrower range, depending on synthesis conditions, post-treatment, and environmental exposure. Furthermore, factors such as partial oxidation, intercalated species, and substrate interactions can significantly modify the local electronic structure and shift the effective work function.

Seokyeong and colleagues fabricated polymer light-emitting diodes (PLEDs) incorporating MXene as the electrode material under alternating current (AC) operation.^153^ The integration of a Ti_3_C_2_T_*x*_ MXene with (3-aminopropyl) triethoxysilane (APTES) resulted in a low surface roughness of 2.7 nm, enabling the PLEDs to exhibit superior mechanical flexibility compared to devices employing other electrodes, such as silver nanowires, carbon nanotubes, and reduced graphene oxide. At an AC frequency of 1 kHz, the MXene-based PLEDs demonstrated a turn-on voltage of 2.1 V, a current efficiency of 7 cd A^−1^, and a luminance of 12 547 cd m^−2^. The transient injection of charge carriers and the alternating modulation of electric polarity are identified as critical factors in mitigating the undesired electrical breakdown of the MXene electrode and the hole-transport layer.

Despite possessing exceptional potential for applications in photonic devices, MXenes remain hindered by several issues that require resolution. Foremost among these is the problem of environmental instability and oxidation, particularly in the case of Ti_3_C_2_T_*x*_ materials where a high density of surface defects and exposed transition metal sites renders the material extremely susceptible to degradation upon exposure to oxygen and moisture. This oxidation process transforms the inherently conductive MXene into semiconducting oxides, creating undesirable scattering centers that degrade the material's plasmonic and intrinsic nonlinear optical properties. Localized heat accumulation during strong light–matter interactions can result in structural destruction or thermal degradation, thereby limiting their long-term operational stability. Beyond these general stability concerns, heterogeneity in surface chemical properties also poses a significant challenge to achieving consistent photonic performance. Moving beyond general stability concerns, the integration of MXene into active photonic devices, such as LEDs and solar cells, presents specific challenges related to interfacial charge dynamics and energy-level alignment. The work function of Ti_3_C_2_T_*x*_ is extremely sensitive to the ratio of surface terminal groups, which can fluctuate significantly during device fabrication. This leads to unpredictable energy-level misalignment at the absorber interface, often resulting in increased non-radiative recombination and a reduction in open-circuit voltage. Furthermore, although MXenes are capable of passivating surface traps in polycrystalline films, their tendency to agglomerate during solution processing can create short-circuit pathways that severely degrade the fill factor and power conversion efficiency.

A fundamental limitation of MXene-based photodetectors arises from the intrinsic conflict between achieving high sensitivity and maintaining ultrafast response. The metallic nature of MXenes leads to substantial dark current, which elevates the noise floor and restricts the achievable detection limit. Although strategies such as Schottky junction engineering and heterostructure design are employed to suppress dark current and enhance carrier separation, their effectiveness is often compromised by Fermi-level pinning, which suppresses the tunability of the Schottky barrier height. Moreover, the abundant surface terminations and structural defects introduce a high density of trap states that, while beneficial for photoconductive gain, give rise to long-lived carrier trapping and persistent photoconductivity. This fundamentally limits the temporal response, creating a bottleneck for applications requiring high-speed photodetection. Addressing this trade-off remains a critical challenge for the practical deployment of MXene-based optoelectronic devices.

## 5. Conclusions and future perspectives

In summary, the emerging family of 2D materials known as Ti_3_C_2_T_*x*_ MXenes has attracted considerable attention for photonic applications owing to the unique combination of metallic conductivity and outstanding electronic and optical properties. These characteristics enable ultrafast carrier dynamics, broadband optical responses, and excellent light–matter interactions, making Ti_3_C_2_T_*x*_ a promising platform for next-generation photonic devices. Among the various applications, nonlinear photonics has emerged as one of the most extensively investigated areas, where Ti_3_C_2_T_*x*_ MXenes serve as efficient saturable absorbers over a broad spectral range. Optimized MXene-based materials have been successfully integrated into both free-space and fiber laser systems using configurations such as saturable absorber mirrors and sandwich-structured ferrules. Notably, near-perfect optical absorption reported for MXene-based photonic devices is primarily achieved through rational structural engineering, including multilayer architectures, porous structures, metasurfaces, and hybrid composites, rather than being an intrinsic property of MXenes alone. Although research on Ti_3_C_2_T_*x*_ MXene-based photonic technologies is still at a relatively early stage, these materials exhibit tremendous potential for future optoelectronic and photonic applications. Continued advances in experimental characterization, theoretical modeling, and multiscale simulations, including DFT, will be essential for accelerating their transition from laboratory research to practical technologies.

First, precise control and understanding of the work function remain critical challenges for the development of Ti_3_C_2_T_*x*_ MXene-based photonic devices. While theoretical studies predict a broad work-function range across different MXene compositions and ideal surface terminations, experimentally fabricated Ti_3_C_2_T_*x*_ MXenes used in practical devices generally exhibit a much narrower work-function range because of mixed surface terminations, structural defects, oxidation, and environmental effects. Consequently, accurate determination and engineering of the work function under realistic device conditions are essential for optimizing energy-level alignment, interfacial charge transfer, and carrier extraction. Moreover, scalable control of surface functional groups during synthesis remains crucial because the electronic and optical properties of Ti_3_C_2_T_*x*_ are highly sensitive to surface chemistry and defect structures. Therefore, further optimization of etching and surface-modification strategies, including both HF-based and fluoride-free methods, will be important for the scalable fabrication of high-quality MXene films on technologically relevant substrates such as Si, ITO, FTO, and flexible polymers.

Second, the plasmonic properties of Ti_3_C_2_T_*x*_ MXenes are strongly influenced by their surface morphology, carrier concentration, electronic structure, and surface chemistry. A deeper understanding of the relationships between dielectric response, interlayer coupling, optical losses, plasmon resonance, and charge-carrier dynamics will provide valuable insights for rationally designing high-performance photonic and optoelectronic devices.

Finally, integrating Ti_3_C_2_T_*x*_ MXenes as carrier transport layers represents a promising direction for advanced photonic and optoelectronic devices, including LEDs, photodetectors, and solar cells. Device performance depends critically on efficient energy-level alignment and rapid interfacial charge transfer between Ti_3_C_2_T_*x*_ and adjacent active materials. Therefore, systematic control of the work function, surface chemistry, wettability, crystal morphology, and interfacial interactions will be essential for maximizing carrier-transport efficiency while minimizing recombination losses. Continued progress in interface engineering and scalable materials processing is expected to further unlock the potential of Ti_3_C_2_T_*x*_ MXenes for next-generation photonic technologies.

## Conflicts of interest

The authors declare that they have no competing financial interests or personal relationships that could have influenced the work reported in this study.

## Supplementary Material

NA-OLF-D6NA00163G-s001

## Data Availability

No new data were used for the research described in the article. Supplementary information (SI) is available. See DOI: https://doi.org/10.1039/d6na00163g.
