# Supplementary material for: Recent advances in Ti3C2Tx MXenes for photonic applications
Source: Nanoscale Adv. 2026 Aug 25;8(20):5795–814. doi: 10.1039/d6na00163g (PMC13573237; doi:10.1039/d6na00163g)
Supplement: Supplementary file 1 [file NA-008-D6NA00163G-s001.pdf]

Supplementary Information (SI) for Nanoscale Advances.
This journal is © The Royal Society of Chemistry 2026

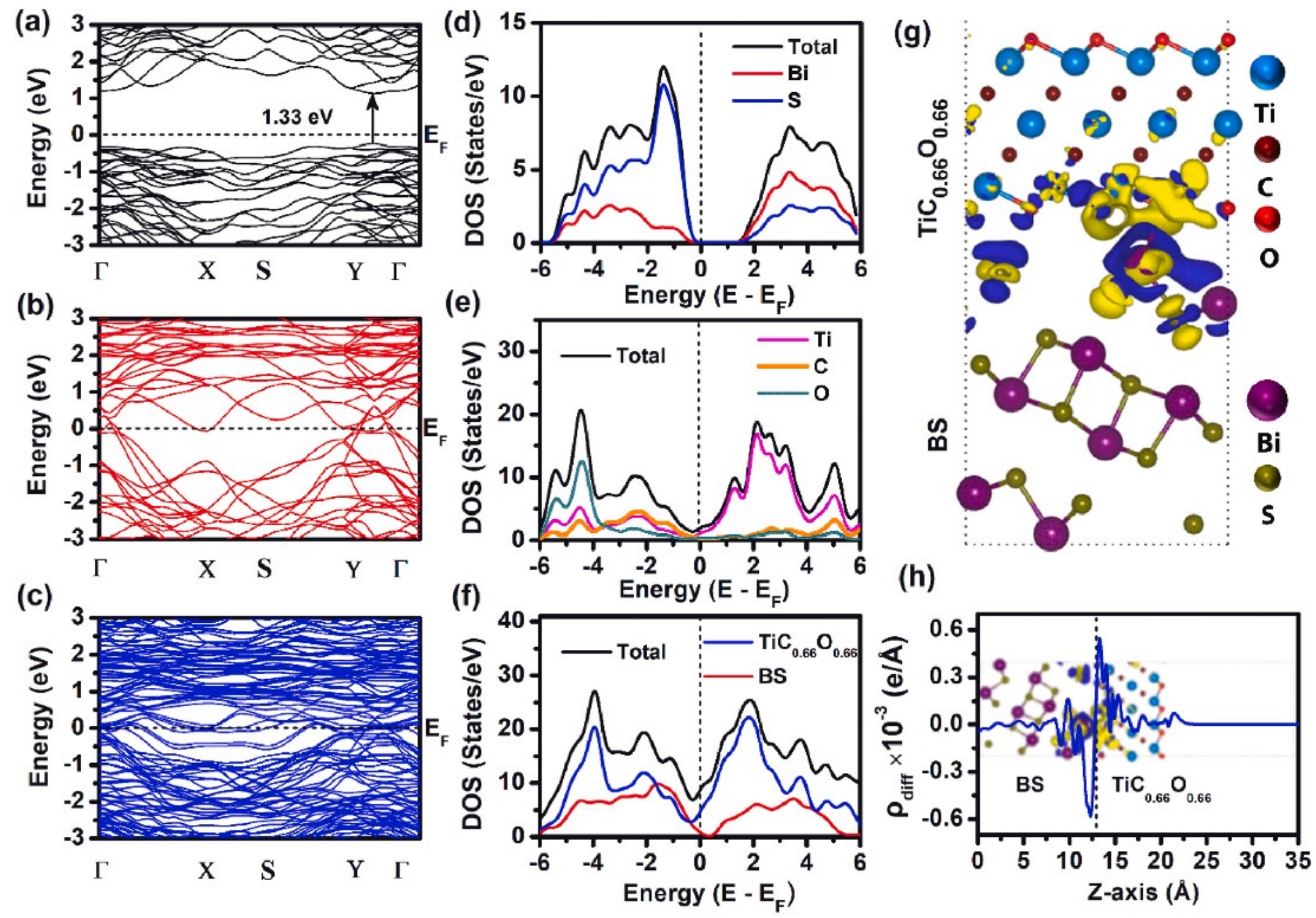

Figure S1. (a–c) Calculated electronic band structures and (d–f) corresponding total density of states (DOS) of pristine $Ti_3C_2T_x$ MXene, Bi_2S_3, and the $Ti_3C_2T_x/Bi_2S_3$ composite. (g, h) Charge density difference plots of the composite, where yellow and blue regions represent charge accumulation and depletion, respectively.[1] This figure has been adapted/reproduced from ref[1] with permission from Elsevier, copyright 2024.

References

1 K. Ghosh and P. K. Giri, *Carbon N. Y.*, 2024, **216**, 118515.
